# Vitamin K_2_ emerges as the key mediator: *Cetobacterium somerae* ZNN-1 increases muscle protein deposition and improves liver health in Nile tilapia (*Oreochromis niloticus*)

**DOI:** 10.1186/s40104-026-01379-x

**Published:** 2026-04-11

**Authors:** Nannan Zhou, Junxi Liu, Xiangfeng Zhang, Guanxiu Xiao, Meiling Zhang

**Affiliations:** 1 Key Laboratory of Microbial Resources Protection, Development and Utilization, College of Biological Sciences and Technology, Yili Normal University, Yining, 835000 China; 2 Lboratory of Aquaculture Nutrition and Environmental Health (LANEH), School of Life Sciences, East China Normal University, Shanghai, 200241 China

**Keywords:** *Cetobacterium somerae*, Lipid catabolism, Nile tilapia, Protein deposition, Vitamin K_2_

## Abstract

**Background:**

*Cetobacterium somerae* (*C. somerae*) is a common indigenous bacterium in the intestine of freshwater fish. Studies have shown that it has the potential to promote protein deposition, but the underlying mechanisms remain unclear.

**Results:**

Nile tilapia were fed with *C. somerae* ZNN-1 (10^8^ CFU/g feed), which significantly increased the carcass ratio, reduced the hepatosomatic index, and decreased whole-body lipid content. Supplementation of *C. somerae* ZNN-1 significantly increased the crude protein content in muscle, promoted glucose uptake and utilization in muscle tissue, and activated the phosphorylation of S6K/S6 in muscle tissue. *C. somerae* ZNN-1 supplementation significantly decreased hepatic total lipid, triglyceride, and free fatty acid contents. Further analysis revealed that *C. somerae* ZNN-1 supplementation markedly activated the phosphorylation of hepatic AMPK and upregulated the expression of genes involved in hepatic lipolysis and fatty acid β-oxidation. Integrated serum metabolomic, bacterial genomic, and gut metagenomic analyses revealed that *C. somerae* ZNN-1 synthesized chorismate (CHA), which serves as a precursor for gut microbiota to produce vitamin K_2_ (VK2). In vitro experiments demonstrated that VK2 activated the S6K/S6 pathway to promote protein synthesis, while stimulating AMPK phosphorylation and activating lipid catabolism to reduce fat accumulation.

**Conclusions:**

These findings provide a theoretical basis for the application of *C. somerae* ZNN-1 in enhancing edible protein content and reducing fat deposition of aquatic animals.

**Supplementary Information:**

The online version contains supplementary material available at 10.1186/s40104-026-01379-x.

## Introduction

Obtaining more edible protein yield, rather than mere weight gain, is a critical yet often overlooked goal in modern aquaculture. There is evidence indicating that excessive weight increase may stem from an increase in the weight of internal organs (such as mesenteric adipose tissue and liver), rather than from a substantial increase in muscle tissue [1–3]. This phenomenon is primarily characterized by excessive accumulation of fat in the mesenteric or liver, which has been identified as a critical risk factor for compromised health in aquatic animals [4–6]. Therefore, a growing number of studies are now focusing on key biometric indexes such as the carcass ratio (carcass is defined as the edible part of the fish body, removed of head, viscera, fins and tail) and hepatosomatic index (HSI), with the aim of conducting more comprehensive assessments of growth and health in aquatic animals [7, 8]. To obtain more edible protein and reduce fat accumulation, feed additives such as bile acids and plant extracts have been widely incorporated into aquatic feeds [9, 10]. Additionally, probiotics have emerged as a green and safe alternative with substantial application potential. Research has demonstrated that dietary supplementation with *Bacillus subtilis* significantly reduced the HSI and decreased hepatic lipid accumulation in grass carp (*Ctenopharyngodon idella*) [11]. Another study on Nile tilapia (*Oreochromis niloticus*) demonstrated that *Bacillus amyloliquefaciens* could increase the CR and enhance the protein content in carcass [7]. However, most probiotics currently used in aquaculture are isolated from fermented foods [12, 13], plants [14], or water bodies [15]. The application of such exogenous strains to farmed fish raises concerns about their safety. For instance, the human-derived probiotic *Lactobacillus rhamnosus* GG has been shown to cause intestinal epithelium injury in zebrafish [16]. Therefore, the development of highly efficient, broadly applicable, and fish-derived probiotics is crucial for advancing sustainable aquaculture.

*Cetobacterium somerae* (*C. somerae*) represents an indigenous bacterium in freshwater fish, which is consistently detected in the intestinal tracts of numerous species including Nile tilapia and grass carp [17, 18]. In recent years, numerous studies have demonstrated that this bacterium can enhance the host's anti-infective ability, improve intestinal health, and affect host metabolism [19–21]. Furthermore, a study has demonstrated that *C. somerae* could enhance the whole-body crude protein content in yellow catfish [22]. Another study on Nile tilapia has shown that *C. somerae* increased CR and reduced the HSI [23]. Despite these notable effects, current research remains confined to phenotypic observations, and the underlying mechanisms by which *C. somerae* promotes protein deposition and reduces lipid content remain to be elucidated. Notably, previous research has identified some key metabolites of *C. somerae* that mediate host physiological regulation; for instance, *C. somerae* can synthesize vitamin B_12_ to enhance the resistance of zebrafish to pathogenic bacterial infections [24], and it can produce argininosuccinic acid to enhance the urea cycle and alleviate ammonia intoxication in yellow catfish (*Pelteobagrus fulvidraco*) [25]. However, more metabolites involved in mediating the interaction between *C. somerae* and the host's protein and lipid metabolism still need to be further identified.

Vitamin K_2_ (VK2) is a fat-soluble vitamin primarily synthesized by microorganisms in nature [26]. Gut microbiota of animals can endogenously synthesize VK2, which is crucial for meeting the host's vitamin K requirements [27]. VK2 exerts significant effects on multiple physiological processes. A recent study revealed that VK2 exhibited positive effects in improving insulin sensitivity, reducing blood glucose levels, and alleviating muscle atrophy [28]. Furthermore, its intake is negatively correlated with the risk of developing metabolic syndrome [29]. However, studies on VK2 in aquatic animals remain scarce, and its role and specific mechanisms in regulating metabolic processes in fish are still unclear.

Nile tilapia is a commercially important aquaculture species, ranking as the third most widely farmed finfish globally [30]. Due to its rapid growth, strong disease resistance, and omnivorous feeding habits, it has become an ideal model for studying metabolic regulation in fish under intensive aquaculture systems [31, 32]. This study employed Nile tilapia as a model to investigate the effects and underlying mechanisms of *C. somerae* ZNN-1, a bacterial strain isolated from the gut of Nile tilapia, on protein deposition and lipid metabolism. The findings aim to provide a theoretical basis for promoting the application of this bacterium in aquaculture.

## Materials and methods

### Bacterial strain and culture conditions

The strain *C. somerae* ZNN-1, isolated from the intestine of Nile tilapia, was streaked on Gifu anaerobic medium (GAM) agar plates to obtain single colonies. It was subsequently identified through 16S rRNA gene sequencing. The *C. somerae* ZNN-1 was cultured in GAM broth medium at 28 °C for 12 h under anaerobic conditions.

### Animal experiments

The juvenile Nile tilapia used in this study were obtained from a commercial hatchery located in Guangzhou, China. A total of 180 fish were randomly allocated into two groups (with three replicate tanks per group, each containing 30 fish). In the 10-week trial, fish in the two groups were respectively fed the control diet (CON) and the control diet supplemented with 10^8^ CFU/g *C. somerae* ZNN-1 (CS). After overnight culture, the *C. somerae* ZNN-1 was washed with PBS and resuspended to an appropriate concentration (10^9^ CFU/mL). The resuspended bacterial suspension was thoroughly mixed with the feed to be fed on the same day at a volume-to-mass ratio of 1 mL:10 g. The control diet was mixed with PBS at the same ratio. The ingredients of the diet were provided in Table S1. During the feeding trial, fish were reared in separate tanks, and 30% water exchange was performed for each tank daily in the morning before feeding, and fish were fed twice daily at 08:00 and 17:00 with a total daily feeding ration equivalent to 4% of their body weight. The total weight of fish in each tank was measured every two weeks to adjust the feeding ration. Water quality was monitored daily. Throughout the experimental period, the water temperature ranged from 26 to 29 °C, dissolved oxygen was maintained between 5.0 and 7.5 mg/L, and pH levels ranged from 7.2 to 7.8, the concentration of ammonia nitrogen was below 0.02 mg/L.

### Sample collection

At the end of the feeding trial, fish were fasted for 24 h prior to sampling. Four fish per tank (i.e., 12 fish per group) were randomly selected and anesthetized using 20 mg/L MS-222 (Western Chemicals Inc., USA). Body weight and body length of each fish were recorded. Blood was then collected from the caudal vein using a 1-mL sterile syringe and centrifuged (4 ºC, 1,000 × *g*, 10 min) to obtain serum, which was stored at −80 °C immediately. Subsequently, the fish were dissected to remove the visceral, and the remaining body was then processed by decapitation, fin removal, and tail removal to obtain the carcass. The liver and carcass of each fish were weighed to calculate the hepatosomatic index (HSI) and carcass ratio (CR). Additionally, muscle and liver tissues were dissected and divided into two portions: one was fixed in 4% paraformaldehyde for subsequent histological analysis, and the other was stored at −80 °C for further analysis. The weight gain, feed conversation ratio, CR and HSI were calculated as follows: weight gain = [(final body weight – initial body weight)/initial body weight] × 100, feed conversion ratio = total feed fed/(total final fish weight – total initial fish weight), CR (%) = (carcass weight/body weight) × 100, HSI (%) = (liver weight/body weight) × 100.

### Proximate composition analysis

Two fish per tank (i.e., six fish per group) were randomly selected for whole-body composition analysis. The feed ingredients were analyzed using standard methods [33]. The protein content in muscle was measured with a Kjeldahl Nitrogen Analyzer (FOSS, Sweden). Total lipid content was determined by chloroform/methanol extraction method [34].

### Biochemical analysis

Hepatic glycogen content, hepatic non-esterified fatty acid (NEFA) content, activities of alanine aminotransferase (ALT) and aspartate aminotransferase (AST) in serum, and glucose content in serum were detected using reagent kits from Nanjing Jiancheng Bioengineering Institute (Cat# A043-1-1, Cat# A042-2-1, Cat#C009-2-1, Cat# C010-2-1, Cat# F006-1-1; Nanjing, China). Hepatic total cholesterol (TC) and triglyceride (TG) levels were measured using commercial reagent kits (Cat# BC1980 and Cat# BC0620; Beijing Solarbio Science & Technology Co., Ltd., Beijing, China).

### Histological analysis

Approximately 1 cm^3^ pieces of liver and muscle tissues were fixed in 4% paraformaldehyde, embedded in paraffin, and subsequently sectioned into 4-μm-thick slices. The sections were stained with hematoxylin and eosin (H&E) and finally photographed using an optical microscope (Nikon Corporation, Japan).

### RNA extraction and gene expression analysis

Approximately 30 mg of liver tissue or 50 mg of muscle tissue were used for RNA extraction with TRIzol reagent. RNA quality was assessed by 1% agarose gel electrophoresis, and concentration was determined using a NanoDrop spectrophotometer. Subsequently, 1 μg of RNA was reverse-transcribed into cDNA using a commercial kit (Cat# R433-01, Vazyme Biotech Co., Ltd., China), and the resulting cDNA was subjected to quantitative real-time PCR (SYBR, Cat# AG11701, Accurate biotechnology (Hunan) Co., Ltd., Changsha, China). The primer sequences were listed in Table S2. The specificity and amplification efficiency of each primer were validated prior to use. The relative expression levels of target genes were calculated using the 2^−^^ΔΔ^^Ct^ method.

### Western blotting (WB)

The detailed protocol for WB was consistent with our previous study [32]. The protein concentration of each sample was determined using a bicinchoninic acid (BCA) assay kit to ensure an equal loading amount of 50 μg of total protein per lane. The information of antibodies used was listed below: CPT1A (Cat# 66039-1-Ig, Proteintech), p-AMPK (Cat# 2535, Cell Signaling Technology), S6K (Cat# 66638-1-Ig, Proteintech), p-S6K (Cat# 28735-1-AP, Proteintech), S6 (Cat# 66886-1-Ig, Proteintech), p-S6 (Cat# 29223-1-AP, Proteintech), GAPDH (Cat# GB15004-100, Servicebio).

### Cell culture and treatments

HepG2 (RRID: CVCL_0027) cells and C2C12 (RRID: CVCL_0188) cells were purchased from Shanghai QuiCell Biotechnology Co., Ltd. (Shanghai, China). HepG2 cells were cultured in MEM medium supplemented with 10% fetal bovine serum and 1% penicillin/streptomycin. C2C12 cells were cultured in DMEM medium containing 10% fetal bovine serum and 1% penicillin–streptomycin.

To investigate the effects of VK2 on lipid metabolism, HepG2 cells were treated with 250 µmol/L oleic acid (OA) in combination with different concentrations of VK2 (5, 10, and 20 µmol/L) for 24 h. Intracellular lipid droplets were detected using a Lipid Droplet Green Fluorescent Assay Kit (Cat# C2053S, Beyotime Biotechnology) according to the instructions. Briefly, the cells were stained with BODIPY 493/503 and Hoechst 33342 at room temperature for 20 min, followed by imaging using a fluorescence microscope (Nikon Ds Ri2, Japan).

To investigate the effects of VK2 on glucose uptake, C2C12 cells were treated with different concentrations of VK2 (5, 10, and 20 µmol/L) for 24 h. The culture supernatant was collected, and the glucose content in the culture supernatant was measured before and after cell treatment. The reduction in glucose content was calculated as the amount consumed by the cells. Subsequently, the cells were harvested for RNA extraction (Cat# RC102-01, Vazyme Biotech Co., Ltd., China) to facilitate subsequent gene expression analysis. Furthermore, cellular glucose uptake capacity was evaluated using a Glucose Uptake Fluorescence Assay Kit with 2-(N-(7-Nitrobenz-2-oxa-1,3-diazol-4-yl) Amino)-2-Deoxyglucose (2-NBDG; Cat# S0561S, Beyotime Biotechnology). Briefly, cells were first subjected to glucose starvation in low-glucose medium for 3 h. Subsequently, they were treated with various concentrations of VK2 (5, 10, and 20 µmol/L) prepared in glucose-free medium for 6 h. Following this, the 2-NBDG glucose working solution was added and the cells were incubated at 37 °C for 30 min. Finally, the nuclei were stained with Hoechst 33342, and imaging was performed using a fluorescence microscope (Nikon Ds Ri2, Japan).

To investigate the effects of VK2 on protein synthesis, C2C12 cells were treated with 10 µmol/L VK2 for 24 h, followed by collecting cell samples for detection of protein expression related to protein synthesis.

### Metabolomics

Metabolite detection in the serum samples from nine fish per group was performed on liquid chromatography-mass spectrometry. The chromatographic column, chromatographic conditions, data preprocessing methods, and statistical analysis procedures were identical to those employed in our previous study [35]. The procedure for statistical screening of metabolites comprised three sequential steps: First, *P*-values were calculated via a *t*-test. Next, dimensionality reduction and derivation of variable importance in projection (VIP) values were performed through orthogonal partial least squares-discriminant analysis (OPLS‑DA) implemented with the R package Ropls. Finally, significant metabolites were identified based on the dual thresholds of *P* < 0.05 and VIP > 1.

### Genomic and metagenomic analyses

Genome extraction, sequencing, assembly, and annotation of *C. somerae* ZNN-1 were performed according to previously published work [36]. The detailed procedures for intestinal content metagenomic DNA extraction, sequencing, and the bioinformatic pipeline (including taxonomic and functional composition analysis) were performed according to our published article [37].

### Data source and acquisition

The raw sequencing data (whole genome of *C. somerae* ZNN-1 and gut metagenome of Nile tilapia) analyzed in this study were obtained from the National Center for Biotechnology Information database under BioProject accession numbers PRJNA1267279 and PRJN1296868.

### Statistical analysis

The normality of data distribution was verified through the Shapiro–Wilk test using GraphPad Prism 9.5 (GraphPad Software, USA). All experimental results are expressed as mean ± standard error of the mean. For comparisons involving two groups, unpaired Student's *t*-tests were utilized. When comparing three or more groups, one-way analysis of variance (ANOVA) with Tukey's multiple comparisons test was employed. Differences were considered statistically significant at *P* < 0.05.

## Results

### Effects of *C. somerae* on the growth and body composition of Nile tilapia

**Fig. 1:**
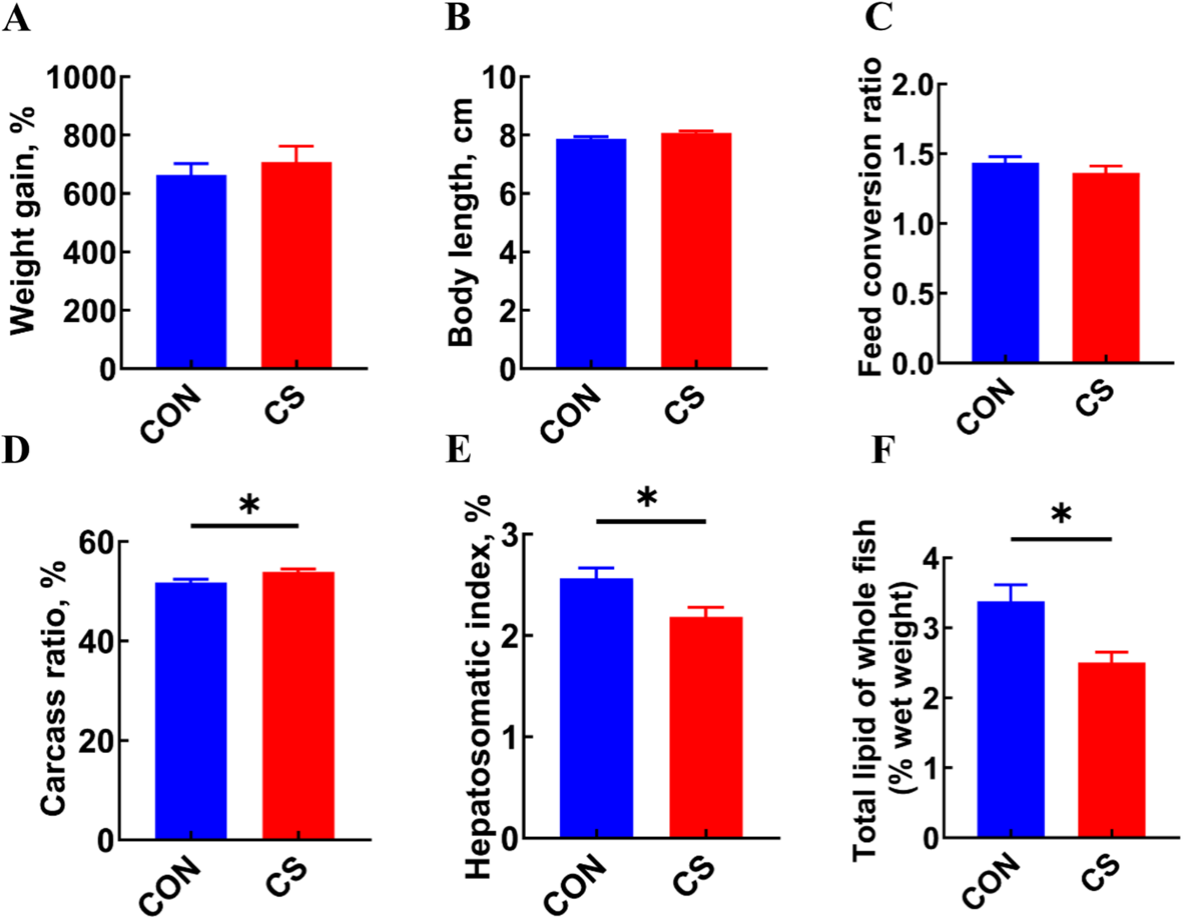
The effects of *C. somerae* ZNN-1 on the growth performance and body composition of Nile tilapia. **A** Weight gain (*n* = 3, replicate tanks). **B** Body length (*n* = 12, replicate fish). **C** Feed conversion ratio (*n* = 3, replicate tanks). **D** Carcass ratio (*n* = 12, replicate fish). **E** Hepatosomatic index (*n* = 12, replicate fish). **F** Total lipid content of whole fish (*n* = 6, replicate fish). ^*^P < 0.05. *P*-value was calculated by Student's *t*-test

After a 10-week feeding trial, the absolute abundance of *C. somerae* in the intestine was significantly higher in the CS group (*P* < 0.05, Fig. S1). The effects of *C. somerae* ZNN-1 on the growth performance of Nile tilapia were evaluated. No significant differences were observed in weight gain, body length, or feed conversion ratio between the two groups of Nile tilapia (*P* > 0.05, Fig. 1A–C). Dietary supplementation with *C. somerae* ZNN-1 significantly increased the carcass ratio (CR) and reduced the hepatosomatic index (HSI) of Nile tilapia (*P* < 0.05, Fig. 1D and E). Furthermore, *C. somerae* ZNN-1 supplementation significantly reduced the total lipid content (*P* < 0.05, Fig. 1F).

### *C. somerae* ZNN-1 promoted muscle protein deposition

**Fig. 2:**
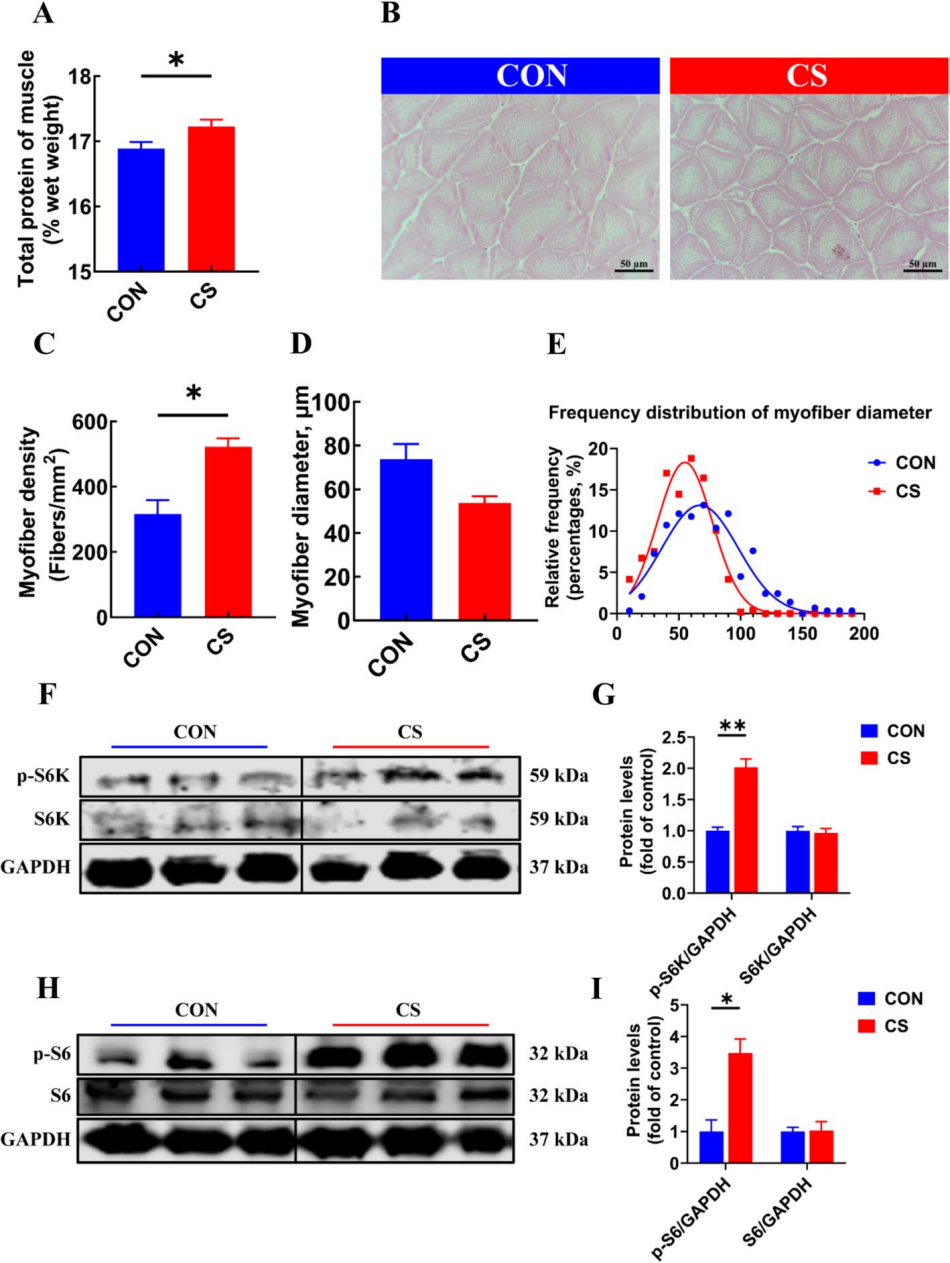
*C. somerae* ZNN-1 promoted muscle protein deposition. **A** Total protein content in muscle (*n* = 12, replicate fish). **B** Hematoxylin and eosin staining of muscle. **C** Myofiber density (*n* = 3, replicate fish). **D** Myofiber diameter (*n* = 3, replicate fish). **E** Frequency distribution of myofiber diameter. **F** Protein expression of p-S6K and S6K (*n* = 3, replicate fish). **G** Protein level of p-S6K and S6K was quantitated and normalized to GAPDH (*n* = 3, replicate fish). **H** Protein expression of p-S6 and S6 (*n* = 3, replicate fish). **I** Protein level of p-S6 and S6 was quantitated and normalized to GAPDH (*n* = 3, replicate fish). ^*^P < 0.05, ^**^P < 0.01, ^***^P < 0.001. *P*-value was calculated by Student's *t*-test

Given that the supplementation with *C. somerae* ZNN-1 significantly increased the carcass ratio (*P* < 0.05, Fig. 1D), we further investigated its effects on protein metabolism. The results showed that fish in the CS group had a higher muscle total protein content (*P* < 0.05, Fig. 2A). Histological analysis with H&E staining revealed that *C. somerae* ZNN-1 supplementation increased the myofiber density in muscle (*P* < 0.05, Fig. 2B and C). No significant difference in myofiber diameter or its frequency distribution was found between the CON and CS groups (*P* > 0.05, Fig. 2D and E). We further detected the phosphorylation levels of S6K and S6, which are key proteins involved in regulating protein synthesis. The results showed that *C. somerae* ZNN-1 significantly increased the levels of phosphorylated S6K and S6 (*P* < 0.05, Fig. 2F–I), but had no effect on their total protein levels (*P* > 0.05, Fig. 2F–I). Taken together, these results suggested that *C. somerae* ZNN-1 promoted muscle protein synthesis by activating the phosphorylation of S6.

### *C. somerae* ZNN-1 enhanced muscle glucose uptake and utilization

**Fig. 3:**
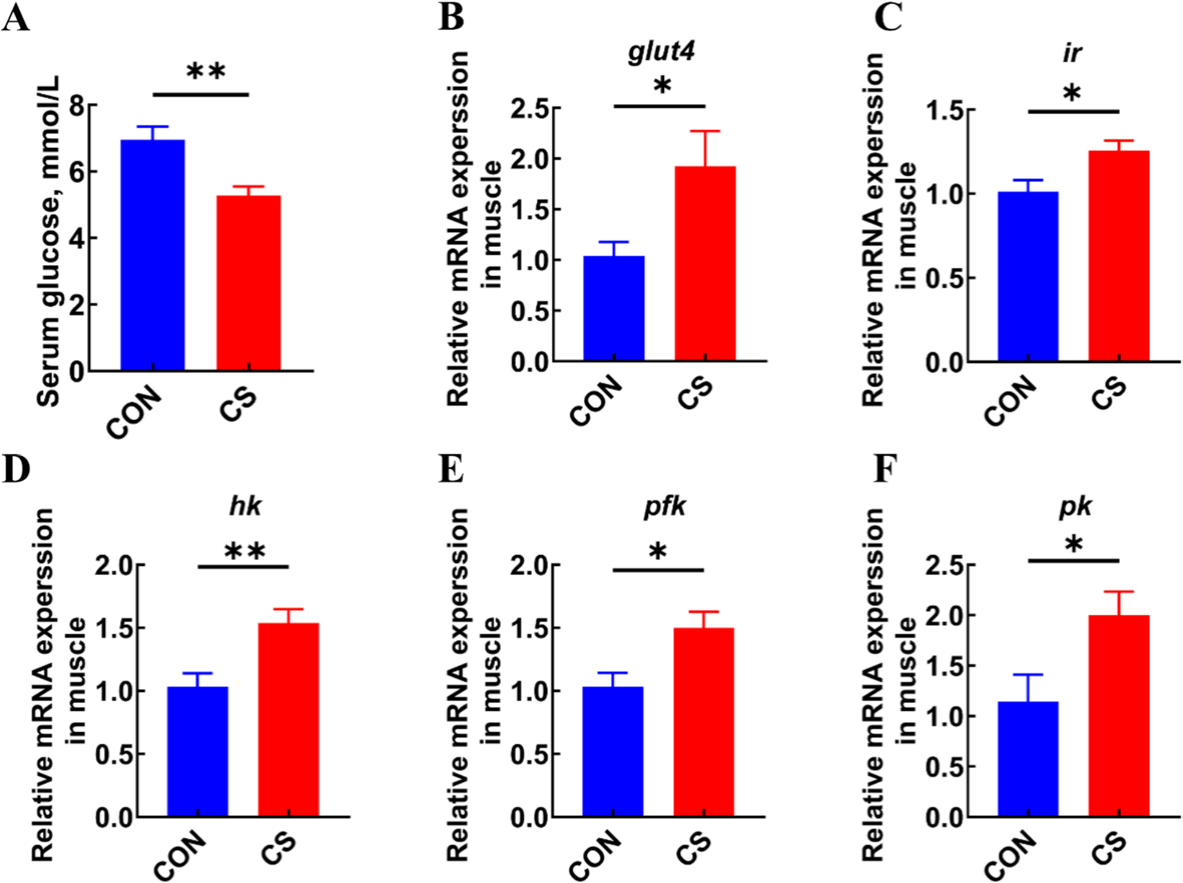
*C. somerae* ZNN-1 enhanced glucose utilization in muscle. **A** Serum glucose (*n* = 12, replicate fish). **B** mRNA expression of *glut4* in muscle (*n* = 6, replicate fish). **C**–**G** mRNA expression of *ir* (**C**), *hk* (**D**), *pfk* (**E**), *pk* (**F**) in muscle (*n* = 6, replicate fish). ^*^P < 0.05, ^**^P < 0.01. *P*-value was calculated by Student's *t*-test

Given that glycolysis can supply both energy and carbon skeletons for protein synthesis [38], we assessed the parameters related to glucose metabolism in muscle tissue. The serum glucose level in fish treated with *C. somerae* ZNN-1 decreased significantly (*P* < 0.05, Fig. 3A). We subsequently measured the gene expression levels of *glut4*, which is the transporter protein responsible for glucose uptake in muscle. The results showed that *C. somerae* ZNN-1 supplementation significantly increased *glut4* expression (*P* < 0.05, Fig. 3B). Given that GLUT4 activation is insulin-dependent, we examined the gene expression of the insulin receptor (*ir*) in muscle. The results showed that the expression of muscle *ir* was significantly increased in the CS group (*P* < 0.05, Fig. 3C). Furthermore, supplementation with *C. somerae* ZNN-1 significantly increased the expression levels of key genes involved in glycolysis (*hk*, *pfk* and *pk*) (*P* < 0.05, Fig. 3D–F). Collectively, our results indicated that *C. somerae* ZNN-1 specifically promoted glucose uptake and utilization in the muscle.

### *C. somerae* ZNN-1 promoted hepatic lipid catabolism and improved liver health

**Fig. 4:**
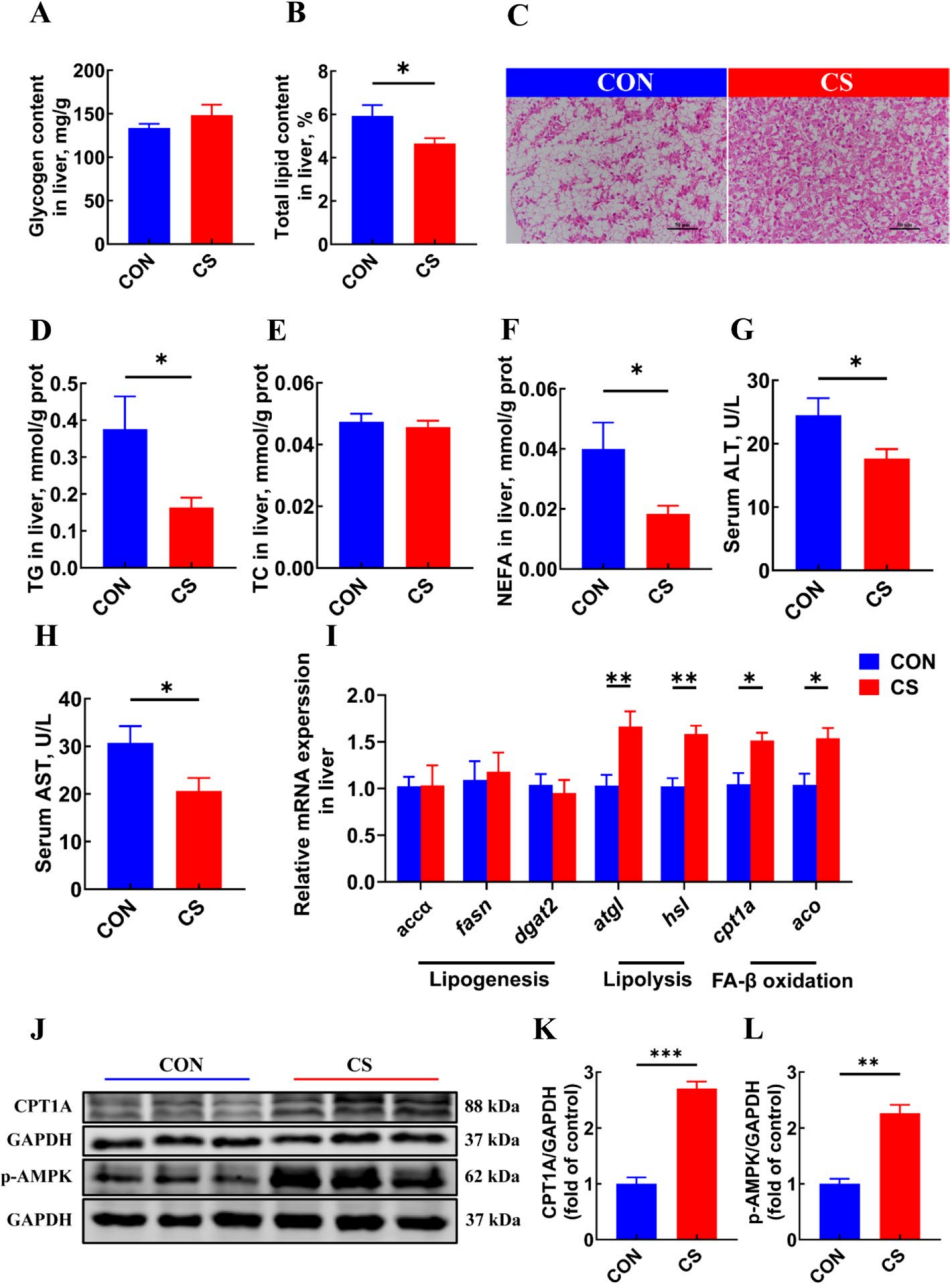
*C. somerae* ZNN-1 reduced liver lipid and improved liver health. **A** Glycogen content in liver (*n* = 12, replicate fish). **B** Total lipid content in liver (*n* = 12, replicate fish). **C** hematoxylin and eosin staining of liver. **D** TG content in liver (*n* = 12, replicate fish). **E** TC content in liver (*n* = 12, replicate fish). **F** NEFA content in liver (*n* = 12, replicate fish). **G** Activity of ALT in serum (*n* = 12, replicate fish). **H** Activity of AST in serum (*n* = 12, replicate fish). **I** Relative mRNA expression of genes related to lipid metabolism in liver (*n* = 6, replicate fish). **J** Protein expression of CPT1A and p-AMPK (*n* = 3, replicate fish). **K** Protein level of CPT1A was quantitated and normalized to GAPDH (*n* = 3, replicate fish). **L** Protein level of p-AMPK was quantitated and normalized to GAPDH (*n* = 3, replicate fish). ^*^P < 0.05, ^**^P < 0.01, ^***^P < 0.001. *P*-value was calculated by Student's *t*-test

Considering the decrease in HSI and total lipid content of whole fish (*P* < 0.05, Fig. 1F and G), liver glycogen and lipid contents were further analyzed. The results showed that *C. somerae* ZNN-1 had no effect on the glycogen content (*P* > 0.05, Fig. 4A). However, it significantly reduced the total lipid content (*P* < 0.05, Fig. 4B). Histological analysis with H&E staining revealed that *C. somerae* ZNN-1 supplementation reduced hepatic vacuolation (Fig. 4C). Moreover, hepatic TG content was significantly decreased in the CS group compared with that in the CON group (*P* < 0.05, Fig. 4D). In contrast, hepatic TC content did not differ between the CON and CS groups (*P* > 0.05, Fig. 4E). Compared with the CON group, hepatic NEFA levels were significantly lower in the CS group of Nile tilapia (*P* < 0.05, Fig. 4F). *C. somerae* ZNN-1 supplementation significantly reduced the serum activity of ALT and AST (*P* < 0.05, Fig. 4G and H). We further investigated the effects of *C. somerae* ZNN-1 on the expression level of genes related to lipid metabolism in the liver. The results indicated that the expression of genes related to lipogenesis, including *accα*, *fasn*, *dgat2*, showed no significant differences between the CON and CS groups (*P* > 0.05, Fig. 4I). However, the addition of *C. somerae* ZNN-1 significantly increased the expression of genes related to lipolysis (including *atgl* and *hsl*) and fatty acid β-oxidation (including *cpt1a* and *aco*) (*P* < 0.05, Fig. 4I). In addition, the protein expression level of CPT1A was significantly increased in the CS group (*P* < 0.05, Fig. 4J and K). To further determine the effects of *C. somerae* ZNN-1 on hepatic energy expenditure, the phosphorylation level of adenosine monophosphate-activated protein kinase (AMPK), a key protein in regulating energy homeostasis, was detected. The result showed that the protein expression level of phosphorylated-AMPK (p-AMPK) was significantly increased in the CS group (*P* < 0.05, Fig. 4J and L). Taken together, these results suggested that *C. somerae* ZNN-1 supplementation promoted lipid catabolism via AMPK activation and improved liver health.

### *C. somerae* ZNN-1 increased serum vitamin K_2_ levels

**Fig. 5:**
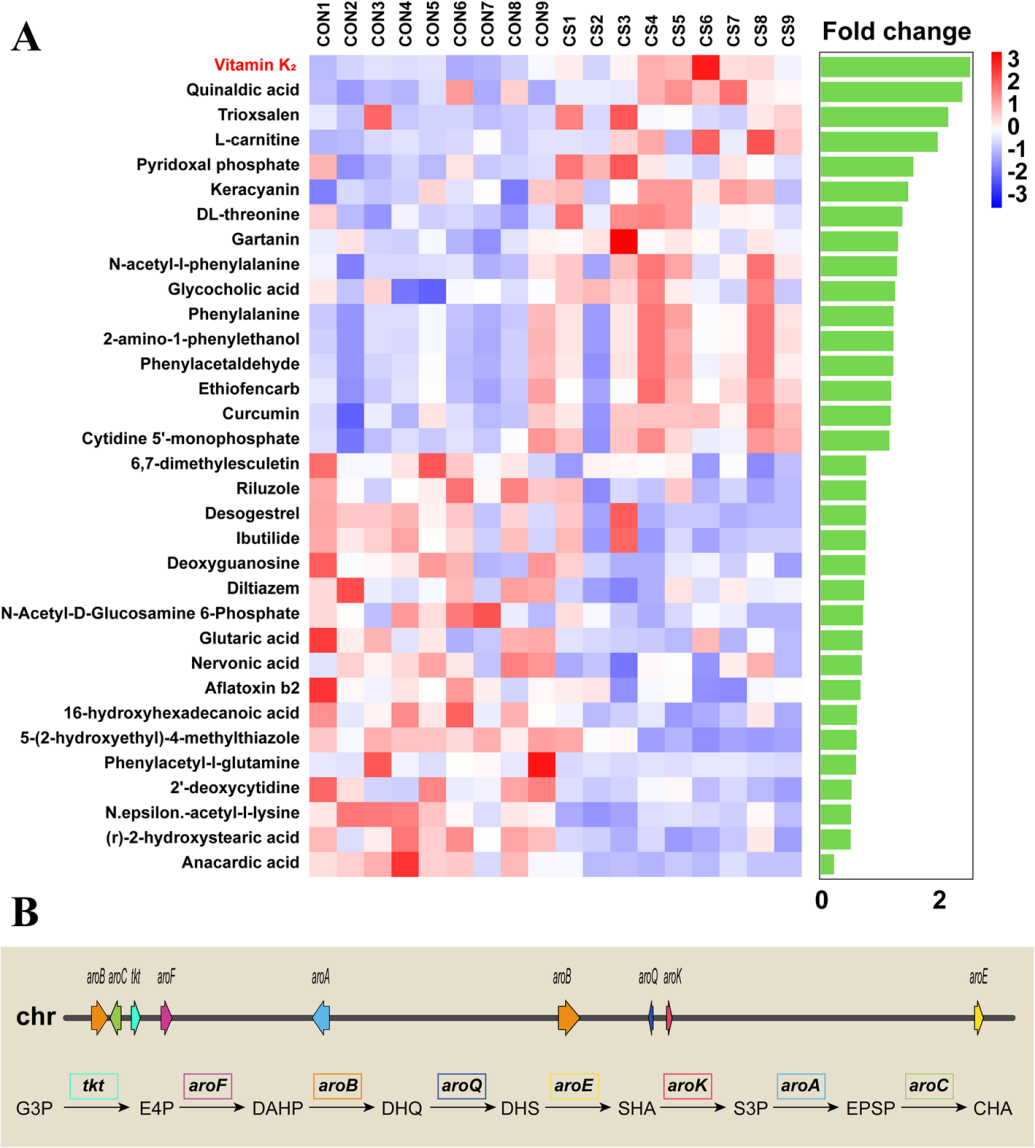
*C. somerae* ZNN-1 enriched serum VK2 content. **A** Interactive heatmap-barplot of significantly differential metabolites between CON and CS groups (*n* = 9). **B** Genomic localization of CHA biosynthesis-related genes

To investigate the metabolites mediating the effects of *C. somerae* ZNN-1 on Nile tilapia, an untargeted metabolomics analysis of serum was performed. The results showed that a total of 33 metabolites in the serum with significant differences were identified between the CON and CS groups. Of these, the level of vitamin K_2_ (*m/z*: 443.30151, rt: 167.98 s) increased significantly in the CS group, and its increase was the most pronounced (Fig. 5A). Through genome-wide analysis, we found that *C. somerae* ZNN-1 possesses a complete set of shikimate pathway genes required for the synthesis of chorismate (CHA), a key precursor of VK2, from the glycolytic intermediate glyceraldehyde-3-phosphate (G3P) (Fig. 5B). Due to the lack of a pure chorismate standard, targeted detection was performed for shikimic acid, a key metabolite in the shikimate pathway, which revealed a significantly increased concentration in the fermentation supernatant of *C. somerae* ZNN-1 (*P* < 0.05, Fig. S2A). The expression of the *aroC* gene, a key gene encoding enzyme for chorismate synthesis in the intestinal microbiota, was significantly up-regulated in the CS group (*P* < 0.05, Fig. S2B). These results indicated that *C. somerae* ZNN-1 can provide the key precursor to facilitate the synthesis of VK2.

### VK2 activated S6K/S6 phosphorylation in C2C12 cells

**Fig. 6:**
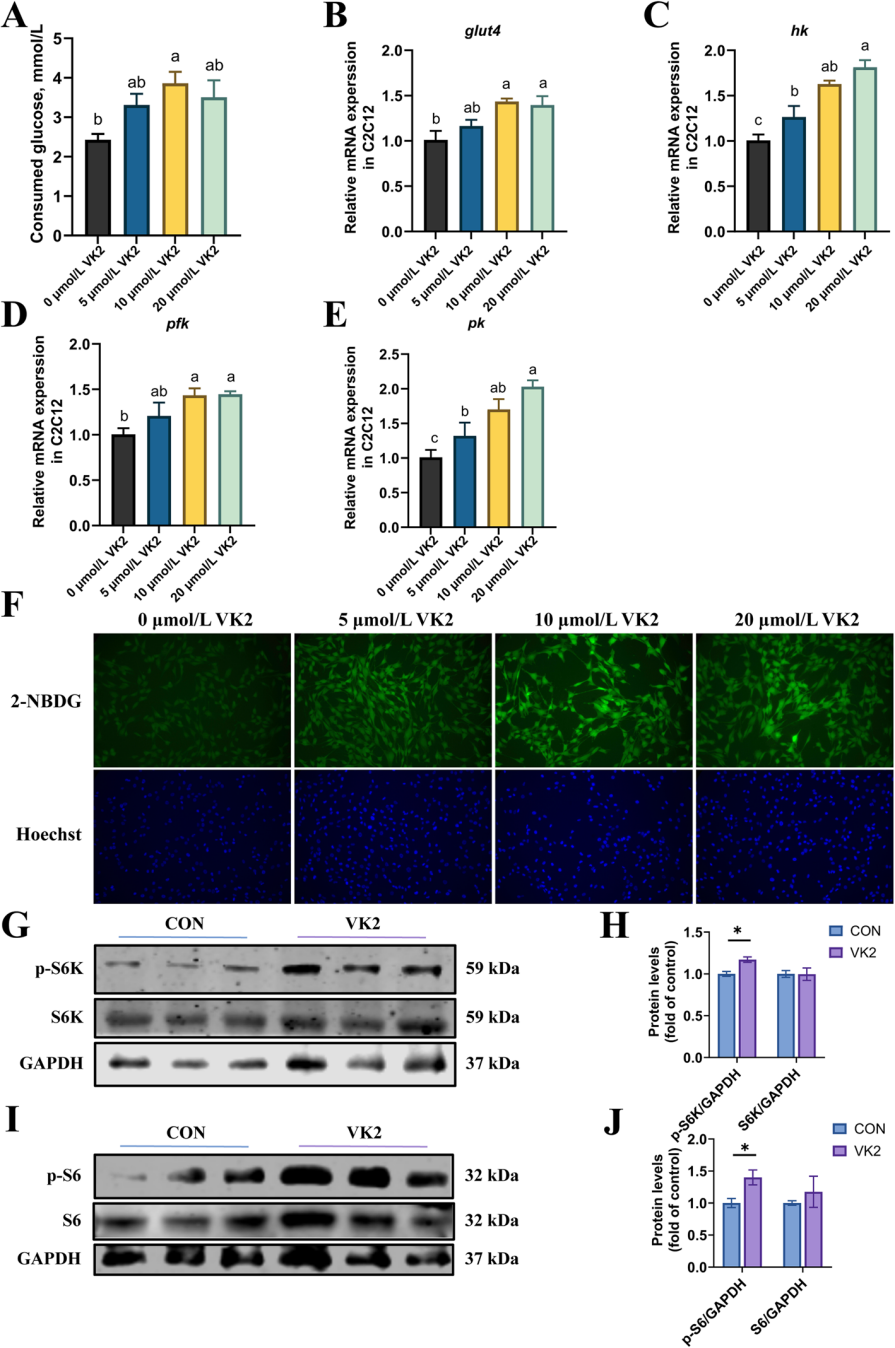
VK2 enhanced glucose uptake and activated S6 phosphorylation in C2C12 cells. **A** Consumed glucose of C2C12 cells (*n* = 3). **B**–**E** mRNA expression of *glut4* (**B**),* hk* (**C**),* pfk* (**D**),* pk* (**E**) in C2C12 cells (*n* = 3). **F** 2-NBDG (green) and Hoechst (blue) were used to stain 2-NBDG-6-phosphate and nuclei, respectively. **G** Protein expression of p-S6K and S6K (*n* = 3). **H** Protein level of p-S6K and S6K was quantitated and normalized to GAPDH (*n* = 3). **I** Protein expression of p-S6 and S6 (*n* = 3). **J** Protein level of p-S6 and S6 was quantitated and normalized to GAPDH (*n* = 3). ^*^P < 0.05. *P*-value was calculated by Student's *t*-test. Data labeled with different superscript letters are significantly different (*P* < 0.05) by one-way ANOVA with Tukey's post-hoc test (**A**–**E**)

To investigate whether VK2 promotes glucose uptake and utilization in muscle cells, C2C12 cells were treated with VK2 at the concentrations of 5 µmol/L, 10 µmol/L, and 20 µmol/L. Exposure of C2C12 cells to 10 µmol/L VK2 resulted in a significant increase in glucose consumption (*P* < 0.05, Fig. 6A). The expression of *glut4* was significantly increased by treatment with 10 µmol/L and 20 µmol/L VK2 (*P* < 0.05, Fig. 6B). Furthermore, VK2 increased the expression of key glycolysis genes (including *hk*, *pfk* and *pk*) in a dose-dependent manner (*P* < 0.05, Fig. 6C–E). The impact of VK2 on glucose uptake was assessed with the fluorescent probe 2-NBDG. A significant increase in the intracellular 2-NBDG content after VK2 treatment was observed in C2C12 cells (Fig. 6F), which indicated that VK2 enhanced glucose uptake capacity in C2C12 cells.

Given that treatment with 10 µmol/L VK2 is sufficient to increase glycolytic flux (*P* < 0.05, Fig. 6C–E), C2C12 cells were exposed to 10 µmol/L VK2 to investigate the regulatory effect of VK2 on the phosphorylation levels of S6K and S6. The results showed that VK2 activated the phosphorylation of S6K and S6 (*P* < 0.05, Fig. 6G–J), without altering the expression levels of their total proteins (*P* > 0.05, Fig. 6G–J). Collectively, these results demonstrated that VK2 can significantly enhance glucose utilization capacity in C2C12 cells, upregulate the phosphorylation level of S6K and S6 to promote protein synthesis.

### VK2 activated AMPK phosphorylation and promoted lipid catabolism in HepG2 cells

**Fig. 7:**
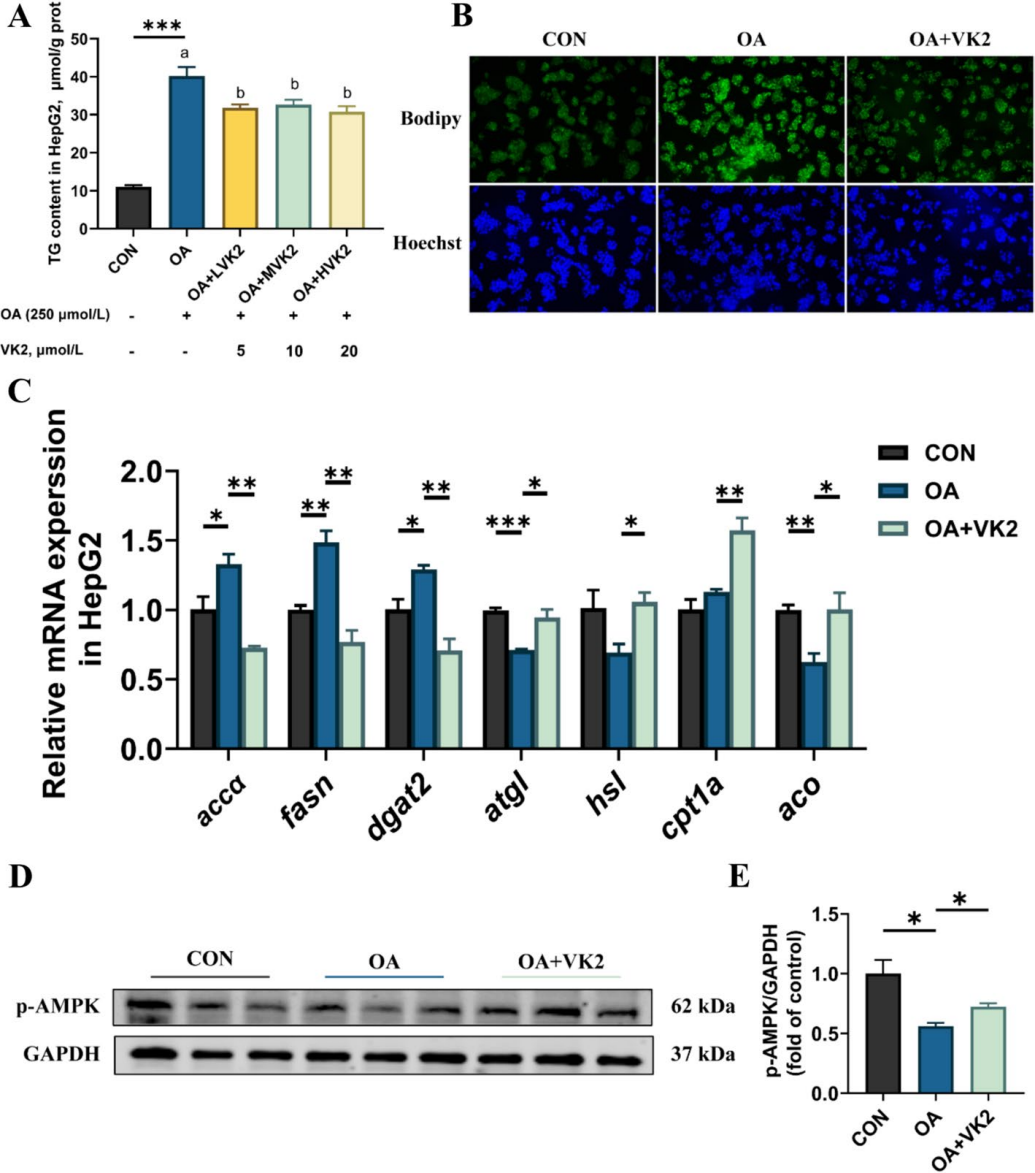
VK2 activated AMPK phosphorylation and promoted lipid catabolism in HepG2 cells. **A** TG content (*n* = 3). **B** BODIPY 493/503 (green) and Hoechst (blue) were used to stain lipid droplets and nuclei, respectively. **C** Gene expression of lipid metabolism (*n* = 3). **D** Protein expression of p-AMPK (*n* = 3). **E** Protein level of p-AMPK was quantitated and normalized to GAPDH (*n* = 3). ^*^P < 0.05, ^**^P < 0.01, ^***^P < 0.001. *P*-value was calculated by Student's *t*-test. Data labeled with different superscript letters are significantly different (*P* < 0.05) by one-way ANOVA with Tukey's post-hoc test (**A**)

To investigate the effect of VK2 on promoting hepatocyte lipid catabolism, HepG2 cells were used to establish a lipid accumulation model by treatment with 250 µmol/L OA. VK2 at concentrations of 5 µmol/L, 10 µmol/L, and 20 µmol/L was added to the oleic acid-treated HepG2 cells. Cellular TG content was significantly increased in the OA group compared with the CON group. However, the treatment with different concentrations of VK2 effectively reduced this accumulation (*P* < 0.05, Fig. 7A). Subsequently, a concentration of 10 µmol/L VK2 was selected for further experimental treatments. Analysis of BODIPY staining revealed that VK2 supplementation significantly reduced the intracellular lipid droplet accumulation induced by OA (Fig. 7B). OA treatment significantly increased the expression levels of lipid synthesis-related genes (including *accα*, *fasn* and *dgat2*), while the addition of VK2 reduced them (*P* < 0.05, Fig. 7C). Conversely, OA down-regulated the expression of genes related to lipolysis (*atgl*) and fatty acid β-oxidation (*aco*), while VK2 supplementation significantly up-regulated the expression levels of *atgl*, *hsl*, *cpt1a* and *aco* (*P* < 0.05, Fig. 7C). Interestingly, OA treatment significantly reduced the phosphorylation level of AMPK in the HepG2 cells, and this effect was reversed by VK2 treatment (*P* < 0.05, Fig. 7D and E). Collectively, these results suggested that VK2 activated AMPK phosphorylation and promoted lipid catabolism.

## Discussion

*C. somerae* is a common indigenous bacterial strain found in various freshwater fish species such as yellow catfish [25], Nile tilapia [18] and grass carp [17]. Numerous studies have demonstrated that *C. somerae* can enhance resistance to infections [39], improve intestinal health [21], and regulate glucose homeostasis [20]. However, its role and underlying mechanisms in enhancing edible protein content and reducing fat deposition in fish remain unclear. In this study, we identified that the indigenous gut bacterium *C. somerae* ZNN-1 promoted muscle protein deposition and alleviated hepatic lipid accumulation in Nile tilapia without affecting overall weight gain. Mechanistically, *C. somerae* ZNN-1 acts by supplying CHA to the gut microbiota, stimulating VK2 synthesis, and subsequently activated S6K/S6 signaling pathway in muscle and AMPK in liver.

Our results indicated that the supplementation of *C. somerae* ZNN-1 at a dosage of 1 × 10^8^ CFU/g of feed has no significant effect on the weight gain of Nile tilapia, which was consistent with previous studies conducted on yellow catfish [22], Nile tilapia [23], and largemouth bass (*Micropterus salmoides*) [40]. However, one study on grass carp demonstrated that adding *C. somerae* at a dosage of 1.35 × 10^9^ CFU/g of feed significantly increased the weight gain rate [21]. Given that grass carp is herbivorous, while Nile tilapia, yellow catfish, and largemouth bass are either carnivorous or omnivorous, and considering the differences in gut microbiota composition among fish with different feeding habits [20], we hypothesized that the differences in the weight-promoting effect of *C. somerae* observed across different studies may be attributed to the differences in gut microbiota composition among fishes with different feeding habits, or the dosage of *C. somerae* used in the experiments.

Although no difference in weight gain was observed, we found that the supplementation of *C. somerae* ZNN-1 significantly increased the CR. CR is used to reflect the edible portion of fish [8]. Muscle constitutes the primary component of the carcass. In our study, we observed that both the CR and muscle protein content significantly increased following the supplementation of *C. somerae* ZNN-1. A previous study showed that *C. somerae* increased the CR and elevated serum amino acid content [23]. Another study in yellow catfish [22] and grass carp [21] reported that *C. somerae* increased the crude protein content of the whole fish. These findings implied that *C. somerae* had the potential to modulate fish protein metabolism. The anabolic metabolism of protein is regulated by mTOR signaling [41]. A study on grass carp demonstrated that the metabolic product acetate of *C. somerae* activated the intestinal Phosphoinositide 3-kinase (PI3K)/protein kinase B (AKT) signaling pathway [21]. Notably, the PI3K/AKT pathway acts as an upstream regulator of mTOR [41]. Consistently, phosphorylation analysis of downstream mTOR signaling effectors revealed that *C. somerae* ZNN-1 significantly activated S6K and S6. In addition to requiring energy and nitrogen sources, protein synthesis also needs carbon skeletons as a structural foundation. Through the glycolysis pathway, glucose not only supplies ATP but also provides the essential carbon molecular skeletons derived from its metabolic intermediates for amino acid synthesis [42]. A study showed that extracellular vesicles promoted amino acid biosynthesis by activating glucose uptake pathway, thereby effectively enhancing the efficiency of protein synthesis [43]. Our results showed that treatment with *C. somerae* ZNN-1 significantly up-regulated the expression of the *glut4* gene, which is responsible for glucose transport in muscle [44], while effectively reducing serum glucose levels, indicating that *C. somerae* ZNN-1 enhanced muscle uptake of serum glucose. GLUT4-mediated glucose uptake is regulated by insulin signaling [45]. Previous studies demonstrated that *C. somerae* elevated serum insulin levels in Nile tilapia and zebrafish [20, 23], and our results revealed that *C. somerae* ZNN-1 increased the expression of insulin receptor (*ir*) in muscle. These findings indicated that *C. somerae* ZNN-1 exerted an activating effect on the insulin signaling pathway. In this study, treatment with *C. somerae* ZNN-1 significantly up-regulated the expression of key glycolysis genes in muscle tissue, indicating that *C. somerae* ZNN-1 further supported amino acid synthesis by enhancing the glycolytic flux in Nile tilapia.

In addition to increasing the CR, we also found that *C. somerae* ZNN-1 reduced the HSI. A decrease in the HSI is often associated with reduced liver fat content [2, 46]. Consistent with a previous study in Nile tilapia [23], our results indicated that the addition of *C. somerae* ZNN-1 significantly reduced the HSI. Observations of liver HE-stained sections, together with measurements of TG content and free fatty acid levels, consistently demonstrated that *C. somerae* ZNN-1 supplementation significantly decreased liver fat content. Analyses of lipid metabolism-related genes further revealed that *C. somerae* ZNN-1 upregulated key genes involved in lipolysis and fatty acid β-oxidation, indicating a significant enhancement of lipid catabolism. A study in common carp (*Cyprinus carpio*) revealed that fermented products of *C. somerae* upregulated the expression of key fatty acid β-oxidation genes, *cpt1a* and *pparα* [47], suggesting that *C. somerae* ZNN-1 may regulate host liver metabolism through its microbial metabolites.

Currently, research in aquatic species remains predominantly focused on a limited spectrum of microbial metabolites, such as short-chain fatty acids and bile acids [1, 48, 49]. Compared with the progress in livestock and poultry, the exploration of microbial metabolite diversity and their functional roles in aquatic species still presents substantial potential for further investigation. Gut microbiota-derived metabolites can influence the metabolism of peripheral organs such as the liver and muscle through the circulatory system [50, 51]. Our study revealed that the serum concentration of VK2 underwent the most pronounced alteration. VK2 is produced by intestinal bacteria [26]. Through genomic analysis of *C. somerae* ZNN-1, we identified that it lacks enzymes directly catalyzing VK2 biosynthesis, while confirming the presence of complete enzymatic pathways for de novo synthesis of its precursor, CHA. Analysis of Nile tilapia gut metagenomic sequencing dataset generated in our other work [37] revealed the presence of key genes responsible for VK2 biosynthesis using CHA as a substrate in the gut microbiome, with expression levels of most genes showing positive correlation with *C. somerae* abundance (Fig. S3). In addition, the expression levels of *menA* and *ubiE*, the genes encoding key enzymes involved in the conversion of chorismate to VK2 in the intestinal microbiota, were significantly increased after the addition of *C. somerae* ZNN-1 (Fig. S4A and B). These findings suggested a cooperative interaction whereby *C. somerae* ZNN-1 supplied the substrate for VK2 production by other gut microbes, forming a metabolic handoff mechanism within the intestinal ecosystem.

VK2 is a fat-soluble vitamin, and its primary function is to maintain bone health [52]. However, recent studies also revealed that this vitamin possessed metabolic regulatory capabilities [28, 53]. A study reported that VK2 ameliorated insulin resistance and activated the AKT/mTOR signaling pathway to attenuate muscle atrophy [28]. Consistent with this, in our study, VK2 significantly increased the phosphorylation of mTOR downstream effectors S6K and S6 in C2C12 cells. Furthermore, we demonstrated that VK2 treatment upregulated *glut4* expression, reduced extracellular glucose levels, and markedly elevated the expression of key glycolysis genes in C2C12 cells. Collectively, these findings suggested that *C. somerae* ZNN-1 likely stimulated insulin signaling through VK2, thereby activating the mTOR pathway while enhancing glucose uptake and utilization in muscle tissue to provide both energy and substrates for protein synthesis.

Beyond its role in protein metabolism, VK2 has also been reported to promote fatty acid β-oxidation and reduce fat deposition [53], consistent with our experimental findings in HepG2 cells demonstrating its efficacy in reducing intracellular lipid accumulation. AMPK serves as a regulator of energy homeostasis, and its phosphorylation effectively promotes catabolism [54]. A study in mice demonstrated that VK2 could activate the phosphorylation of AMPK [26]. Consistent with this, we found that VK2 enhanced AMPK phosphorylation in HepG2 cells and promoted lipid catabolism. These findings collectively indicated that *C. somerae* ZNN-1 facilitates VK2 synthesis by the gut microbiota through substrate provision, thereby activating hepatic AMPK phosphorylation to promote lipid catabolism, and ultimately reducing hepatic fat content and improving liver health. In the present study, validation of VK2’s effects relied on in vitro experiments with mammalian cell lines. These results represent only preliminary mechanistic clues and cannot fully reflect the true physiological reality in fish. Further validation of this hypothesis via in vivo experiments in fish will be performed in future work.

## Conclusion

**Fig. 8:**
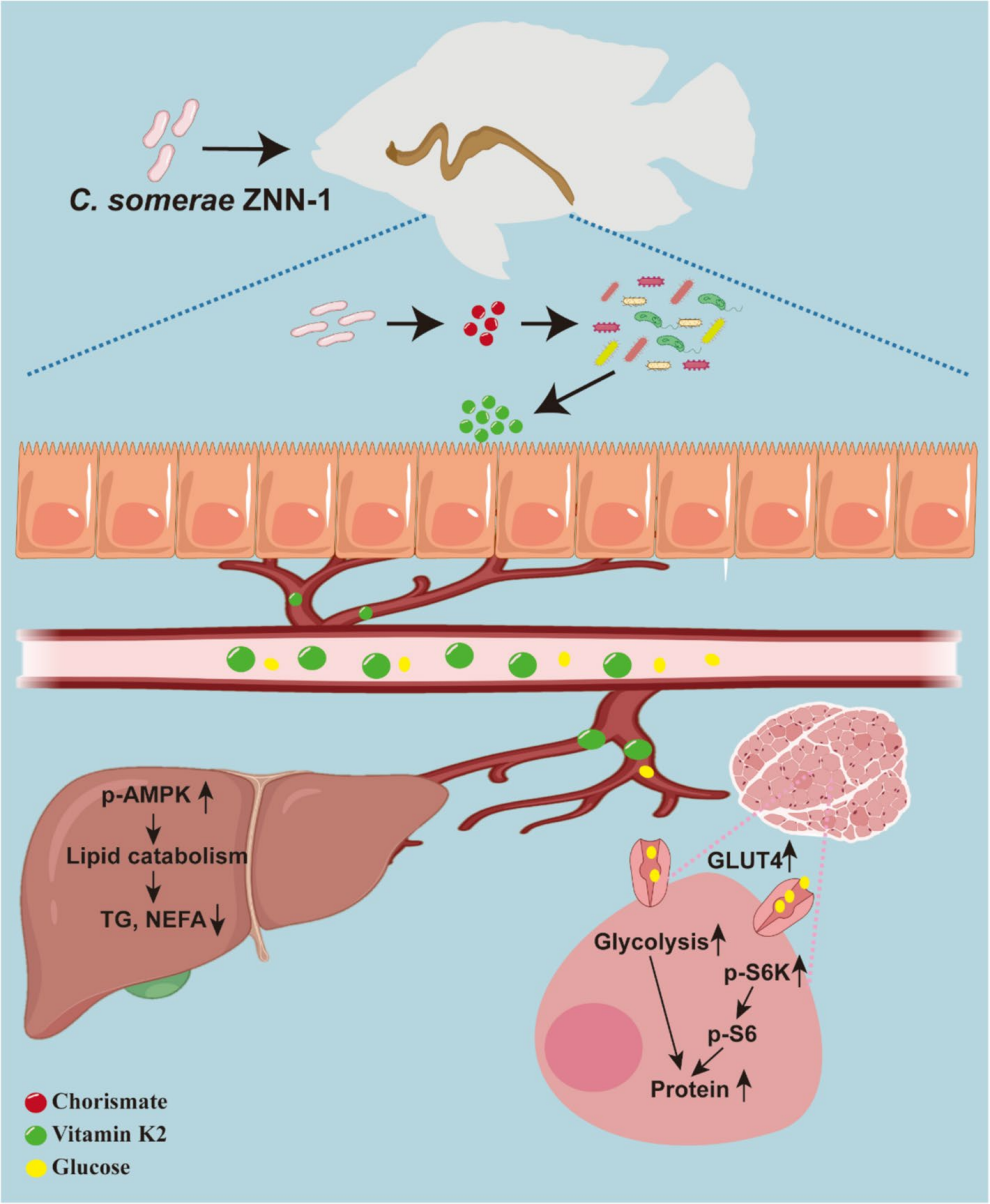
Potential mechanism of *C. somerae* ZNN-1 in promoting muscle protein deposition and improving liver health in Nile tilapia

In summary, the supplementation of *C. somerae* ZNN-1 at 10^8^ CFU/g feed did not affect the weight gain of Nile tilapia but promoted muscle protein deposition and improved liver health. To our knowledge, this study provides the first elucidation of the mechanism by which *C. somerae* promoted protein deposition and improved liver health in fish. As illustrated in Fig. 8, *C. somerae* ZNN-1 synthesized CHA, providing a substrate for the gut microbiota to synthesize VK2. VK2 activated hepatic AMPK to promote lipid catabolism and thereby improved liver health. Additionally, VK2 enhanced glucose uptake and utilization in muscle and activated the mTOR/S6K/S6 signaling pathway to promote protein deposition. This study established a theoretical foundation for the application of indigenous strain *C. somerae* in enhancing edible protein content and reducing fat deposition of aquatic animals.

## Supplementary Information

Additional file 1: Supplementary methods. Absolute quantitative q‑PCR analysis of *Cetobacterium somerae*; RNA extraction and gene expression analysis of intestinal content microbiota; Quantification of shikimic acid content in the fermentation supernatant. Table S1. Ingredients and proximate chemical composition of the diet of the first experiment. Table S2. Primer sequences. Table S3. Primer sequences of microbial genes in intestinal content. Fig. S1. Absolute quantitative analysis of C. somerea in intestine. Fig. S2. Determination of shikimic acid content and relative mRNA expression of gene related to chorismite biosynthesis. Fig. S3. Microbial biosynthetic pathway of vitamin K_2_ from chorismate. Fig. S4. Relative mRNA expression of genes related to VK2 biosynthesis.

Additional file 2. Full uncropped Gels and Blots images.

## Data Availability

No datasets were generated or analysed during the current study.

## Abbreviations

2-NBDG: 2-(N-(7-Nitrobenz-2-oxa-1,3-diazol-4-yl) Amino)-2-deoxyglucose
*accα*: Acetyl-CoA carboxylase alpha
*aco*: Acyl-CoA oxidase
*atgl*: Adipose triglyceride lipase
AKT: Protein kinase B
ALT: Alanine aminotransferase
AST: Aspartate aminotransferase
ATP: Adenosine triphosphate
AMPK: Adenosine 5′-monophosphate (AMP)-activated protein kinase
ANOVA: Analysis of variance
BCA: Bicinchoninic acid
CON: Control diet
CHA: Chorismate
CPT1A: Carnitine palmitoyltransferase 1A
CR: Carcass ratio
CS: Control diet supplemented with 10^8^ CFU/g *C. somerae* ZNN-1
*C. somerae*: *Cetobacterium somerae*
*dgat2*: Diacylglycerol O-acyltransferase 2
*fasn*: Fatty acid synthase
G3P: Glyceraldehyde-3-phosphate
GAM: Gifu anaerobic medium
GAPDH: Glyceraldehyde-3-phosphate dehydrogenase
*glut4*: Glucose transporter 4
H&E: Hematoxylin and eosin
*hk*: Hexokinase
*hsl*: Hormone-sensitive lipase
HSI: Hepatosomatic index
*ir*: Insulin receptor
mTOR: Mammalian target of rapamycin
NEFA: Non-esterified fatty acid
OA: Oleic acid
p-AMPK: Phospho-adenosine 5′-monophosphate (AMP)-activated protein kinase
*pfk*: Phosphofructokinase
*pk*: Pyruvate kinase
PI3K: Phosphoinositide 3-kinase
p-S6: Phospho-ribosomal S6
p-S6K: Phospho-ribosomal S6 kinase
S6K: Ribosomal S6 kinase
S6: Ribosomal S6
TC: Total cholesterol
TG: Triglyceride
VK2: Vitamin K_2_
WB: Western blotting
