# Supplementary material for: Vitamin K2 emerges as the key mediator: Cetobacterium somerae ZNN-1 increases muscle protein deposition and improves liver health in Nile tilapia (Oreochromis niloticus)
Source: J Anim Sci Biotechnol. 2026 Apr 11;17:64. doi: 10.1186/s40104-026-01379-x (PMC13069694; doi:10.1186/s40104-026-01379-x)
Supplement: Supplementary file 1 — Additional file 1: Supplementary methods. Absolute quantitative q‑PCR analysis of Cetobacterium somerae; RNA extraction and gene expression analysis of intestinal content microbiota; Quantification of shikimic acid content in the fermentation supernatant. Table S1. Ingredients and proximate chemical composition of the diet of the first experiment. Table S2. Primer sequences. Table S3. Primer sequences of microbial genes in intestinal content. Fig. S1. Absolute quantitative analysis of C. somerea in intestine. Fig. S2. Determination of shikimic acid content and relative mRNA expression of gene related to chorismite biosynthesis. Fig. S3. Microbial biosynthetic pathway of vitamin K2 from chorismate. Fig. S4. Relative mRNA expression of genes related to VK2 biosynthesis. [file 40104_2026_1379_MOESM1_ESM.docx]

**Vitamin K2 emerges as the key mediator: Cetobacterium somerae ZNN-1 increases muscle protein deposition and improves liver health in Nile tilapia (Oreochromis niloticus)**

Nannan Zhou^1,2^, Junxi Liu^2^, Xiangfeng Zhang^1^, Guanxiu Xiao^1^, Meiling Zhang^1, 2*^

^1^*Key Laboratory of Microbial Resources Protection, Development and Utilization, College of Biological Sciences and Technology, Yili Normal University, Yining, 835000, China*

*^2^Lboratory of Aquaculture Nutrition and Environmental Health (LANEH),* *School of Life Sciences, East China Normal University, Shanghai 200241, China;*

**Supplementary Methods**

**Absolute quantitative q‑PCR analysis of *Cetobacterium somerae*.**

To quantify the abundance of *Cetobacterium somerae* in fish intestine, six individuals were randomly chosen from each treatment, and intestinal microbial DNA was extracted. A species-specific fragment of *Cetobacterium* *somerae* was amplified from its genomic DNA, purified, and cloned into the pMD19-T vector. A standard curve was established using serially diluted recombinant plasmid to calculate the copy number of the target gene.

**RNA extraction and gene expression analysis of intestinal content microbiota**

Approximately 100 mg intestinal content was used for RNA extraction with magnetic bead-based fecal RNA extraction kit (Guangzhou Magen Biotechnology Co., Ltd, China, CAT#R6626-01). RNA quality was assessed by 1% agarose gel electrophoresis, and concentration was determined using a NanoDrop spectrophotometer. Subsequently, 1 μg of RNA was reverse-transcribed into cDNA using a commercial kit (Vazyme Biotech Co., Ltd, CAT# R433-01, China), and the resulting cDNA was subjected to quantitative real-time PCR (SYBR, Accurate biotechnology (Hunan) Co.,ltd, ChangSha, China, CAT#AG11701). The primer sequences were listed in Table S3. The specificity and amplification efficiency of each primer were validated prior to use. The relative expression levels of target genes were calculated using the 2^(−^ΔΔ^Ct) method.

**Quantification of shikimic acid content in the fermentation supernatant**

Shikimic acid content in the fermentation supernatant of *Cetobacterium somerae* ZNN-1 was analyzed using ultra-high-performance liquid chromatography-quadrupole linear ion trap mass spectrometry (Personalbio, China). Metabolites were extracted using cold methanol/acetonitrile (1:1, v/v) with stable isotope-labeled internal standards. The mixture was centrifuged (14,000 g, 20 min, 4 ℃), and the supernatant was dissolved and reconstituted in 100 μL of acetonitrile/water (1:1, v/v) for further analysis by UHPLC-QTRAP MS. The chromatographic columns utilized were ACQUITY UPLC BEH Amide 1.7 μm, 2.1 mm× 100 mm column and ACQUITY UPLC BEH C18 1.7 μm, 2.1 mm× 100 mm column (Waters, America). Mass spectrometry data acquisition was performed using an AB 6500+ QTRAP mass spectrometer (AB SCIEX, America). MultiQuant was used for quantitative data processing.

**Supplementary Tables**

Table S1. Ingredients and proximate chemical composition of the diet of the first experiment.

| **Ingredients** | **CON** | **CS** |
| --- | --- | --- |
| Soybean meal (g/kg) | 395 | 395 |
| Casein (g/kg) | 160 | 160 |
| Gelatin (g/kg) | 40 | 40 |
| pre-gelatinized starch (g/kg) | 275 | 275 |
| Soybean oil (g/kg) | 38 | 38 |
| Fish oil (g/kg) | 22 | 22 |
| Vitamin premix^1^ (g/kg) | 12 | 12 |
| Mineral premix^2^ (g/kg) | 12 | 12 |
| Ca(H_2_PO_4_)_2_ (g/kg) | 10 | 10 |
| Carboxymethyl cellulose (g/kg) | 25 | 25 |
| cellulose (g/kg) | 3.75 | 3.75 |
| Choline chloride (g/kg) | 5 | 5 |
| Dimethyl-beta-propiothetin (g/kg) | 2 | 2 |
| Butylated hydroxytoluene (g/kg) | 0.25 | 0.25 |
| *C. somerae* (CFU/g) | 0 | 10^8^ |
| Total | 1000 | 1000 |
| Proximate composition, g/kg dry matter |  |  |
| Crude protein | 39.5 | 39.5 |
| Crude lipid | 5.6 | 5.6 |

^1^ Vitamin premix (mg or IU/kg): 500 000 IU vitamin A, 5000 mg vitamin B1, 5000 mg vitamin B2, 5000 mg vitamin B6, 5000 mg vitamin B12, 10 000 mg vitamin C, 50 000 IU vitamin D3, 2500 mg vitamin E, 1000 mg vitamin K3, 250 mg biotin, 100 000 mg cholin, 1000 mg folic acid, 25 000 mg inositol, 25 000 mg niacin, 10 000 mg pantothenic acid.

^2^ Mixed mineral (g/kg): 0.02 g NaSeO_3_, 0.06 g NH_4_ molybdate, 0.08 g CoCl_2_·6H_2_O, 0.16 g KI, 0.62 g CuSO_4_•5H_2_O, 3.12 g MnSO_4_•H_2_O, 4.67g ZnSO_4_·7H_2_O, 10.9 g Fe (II) gluconate, 49.8 g NaCl, 147.4 g MgSO_4_•7H_2_O.

Table S2. Primer Sequences.

| **species** | **Gene** | **Forward primer (5’ to 3’)** | **Reverse primer (5’ to 3’)** | | **GenBank NO** |
| --- | --- | --- | --- | --- | --- |
| Nile tilapia | *β-actin* | AGCCTTCCTTCCTTGGTATGGAAT | TGTTGGCGTACAGGTCCTTACG | | XM_003443127.5 |
|  | *accα* | TAGCTGAAGAGGAGGGTGCAAGA | AACCTCTGGATTGGCTTGAACA | | XM_005471970 |
|  | *fasn* | TCATCCAGCAGTTCACTGGCATT | TGATTAGGTCCACGGCCACA | | [XM_003454056.5](https://www.ncbi.nlm.nih.gov/entrez/viewer.fcgi?db=nucleotide&id=1434946517) |
|  | *dgat2* | GCTTGAATTCTGTCACCCTGAAGA | ACCTGCTTGTAGGCGTCGTTCT | | XM_003458972 |
|  | *atgl* | GACACATGCTGCAAAGCACT | ACCAGGACGTTTTCTCCGTC | | XM_003440346.5 |
|  | *hsl* | AGTTCACTCCAGCCATTCGG | TGGCTGCTACCCCTATTCCT | | XM_005463937.4 |
|  | *cpt1a* | TTCGTGTTTGAGTTGCACGG | CGAAGAGCCTCATGGGACAG | | [XM_003440355.5](https://www.ncbi.nlm.nih.gov/entrez/viewer.fcgi?db=nucleotide&id=1434943577) |
|  | *aco* | AGTCCCACTGTGAGCTCCATCAA | CAGACCATGGCAGTTTCCAAGA | | [XM_003447910.5](https://www.ncbi.nlm.nih.gov/entrez/viewer.fcgi?db=nucleotide&id=1434927097) |
|  | *glut2* | CATTGGCATTCTAATCAGCCAGGT | TTGTAATATTGCTGGCGCTCCA | | XM_003442884.5 |
|  | *glut4* | GCAGGAGGAAAGCCATGCTTATA | ATCATTTCAAAGGAGCGGCAGA | | XM_003458705.4 |
|  | *ir* | CGACCTGACGCCGAGAATAA | GCCAAGTCTCTGTGGACGAA | | XM_005476595.4 |
|  | *hk* | CAGCGGGTCCGAGTTTT | TCAAGCTGAGTGAGGGTTGG | | XM_019365942.2 |
|  | *pfk* | AACCTGTGTGTGATTGGAGGTGAT | CGTGATCTTACCGGCTTTAACAAG | | XM_003441476.5 |
|  | *pk* | ATGGGCAGTGGGAGTAGCA | ATCGAGAGGCTGGCCCAAT | | XM_003437654.5 |
|  | *gys* | CCTCACTCTGCGCTGTTATTC | CAGCGGCATGCCTTCAGTTT | | XM_013276796.3 |
|  | *gp* | ACCAACGGCATTACTCCTCG | CTCCCCAATTCTCTCGGCAA | | XM_003446761.4 |
| human | *β-actin* | CTCCATCCTGGCCTCGCTGT | GCTGTCACCTTCACCGTTCC | | NM_001101.5 |
|  | *accα* | ATGTCTGGCTTGCACCTAGTA | CCCCAAAGCGAGTAACAAATTCT | | XM_047435881.1 |
|  | *fasn* | GGAAGCTGCCAGAGTCGGAGAACT | TGAGGGTCCATCGTGTGTGCCT | | XM_054315477.1 |
|  | *dgat2* | GGCAGGCAACTTCCGAATGCCT | AAAGCCCTTGCGGTTCCGCA | | NM_005172.2 |
|  | *atgl* | CAACATCACCGCAGATCCCA | GAACTGCTCCTCCGACATGG | | NM_001253891.2 |
|  | *hsl* | GAAGGCTATGTTGTCCTCCG | ATGAGAAAACCAGTGCTCGG | | XM_005258937.4 |
|  | *cpt1a* | TCCAGTTGGCTTATCGTGGTG | TCCAGAGTCCGATTGATTTTTGC | | XM_054367696.1 |
|  | *aco* | ACTCGCAGCCAGCGTTATG | AGGGTCAGCGATGCCAAAC | | NM_007292.6 |
| mouse | *β-actin* | GTGACGTTGACATCCGTAAAGA | GCCGGACTCATCGTACTCC | | NM_007393.5 |
|  | *glut4* | AATGTCTTGGCCGTGTTGGG | CCCTGATGTTAGCCCTGAGTAG | [NM_009204.3](https://www.ncbi.nlm.nih.gov/entrez/viewer.fcgi?db=nucleotide&id=2726154829) | |
|  | *hk* | CCAAGTGCAGAAGGTTGACCA | GTCACCCTTACTCGGAGCAC | NM_013820.4 | |
|  | *pfk* | GGCCAAAGGTCAGATTGAGGA | AGCCCCCAATGATGACAAGG | NM_001163487.1 | |
|  | *pk* | CATGCAGCACCTGATAGCTCG | CTCCATGAGGTCTGTGGAGTG | NM_001253883.2 | |
| *Cetobacterium somerae* | | GGCAGTACAGAGAGTTGCCAAC | ACGGTTAGGCCTGCTACTTCA |  | |

*β-actin,* beta-actin; *accα,* acetyl-CoA carboxylase alpha; *fasn,* fatty acid synthase; *dgat2,* diacylglycerol O-acyltransferase 2; *atgl,* adipose triglyceride lipase; *hsl,* hormone-sensitive lipase; *cpt1a,* carnitine palmitoyl transferase 1a; *aco,* acyl-CoA oxidase; *glut2,* glucose transporter 2; *glut4,* glucose transporter 4; *ir,* insulin receptor; *hk,* hexokinase; *pfk,* phosphofructokinase; *pk*, pyruvate kinase; *gys*, glycogen synthase; *gp*, glycogen phosphorylase.

Table S3. Primer sequences of microbial genes in intestinal content.

| **Gene** | **Forward primer (5’ to 3’)** | **Reverse primer (5’ to 3’)** |
| --- | --- | --- |
| *rpoB* | GCCTCTGGCATTTCTTCACC | GGAGAGATGGAGGTTTGGGC |
| *aroC* | CTGGACATGCAGATTTTGCCG | CCAGAAAAATGACCTCCACCT |
| *menA* | GGCAATCGGTGTTCTTGCTC | TAGGTTGTTCCCATCGTCGC |
| *ubiE* | GCTGGTGGTGTGTGAGTTCT | CATCCGGGTTACTGGACACC |

*rpoB:* *RNA polymerase beta subunit; aroC: chorismate synthase; menA, 1,4-dihydroxy-2-naphthoate prenyltransferase; ubiE, ubiquinone/menaquinone methyltransferase.*

**Supplementary Figure**


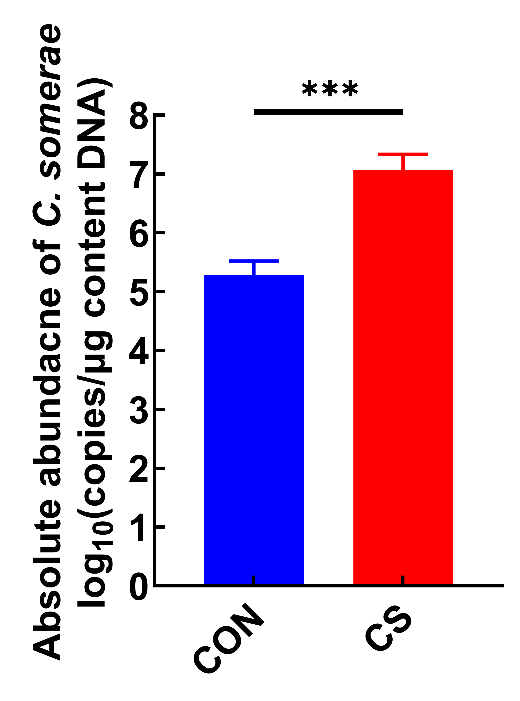


Figure S1. Absolute quantitative analysis of *C. somerea* in intestine. Data was expressed as mean ± SEM (n = 6). *** *P*<0.001. *P*-value was calculated by Student's t-test.


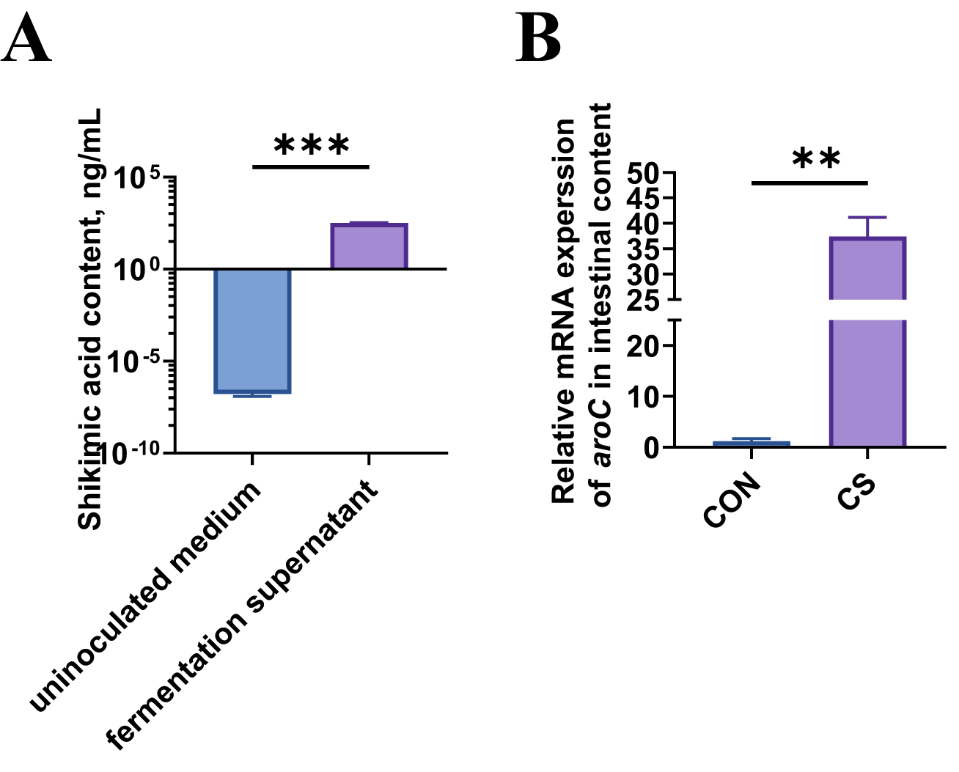


Figure S2. Determination of shikimic acid content and relative mRNA expression of gene related to chorismite biosynthesis. (A) Shikimic acid content in uninoculated medium and *C. somerae* ZNN-1 fermentation supernatant (n=3). (B) mRNA expression of *aroC* in intestinal content of nile tilapia from the CON and CS groups (n=3). ** *P*<0.01, *** *P*<0.001. *P*-value was calculated by Student's t-test.


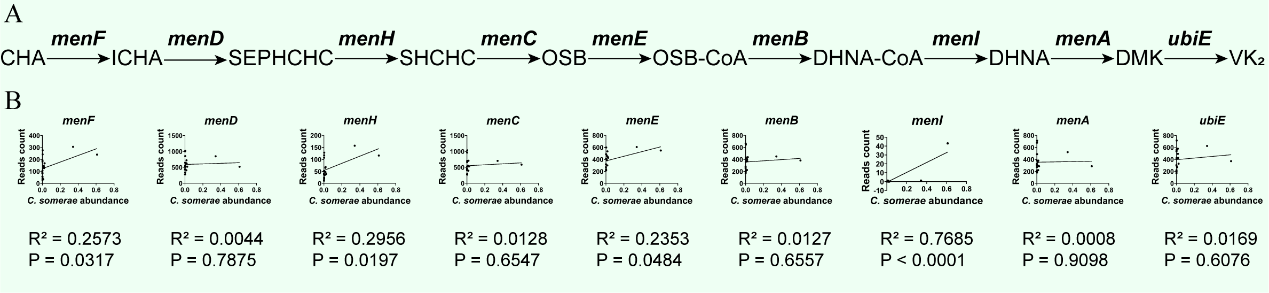


Figure S3 Microbial biosynthetic pathway of vitamin K2 from chorismate. (A) Microbial biosynthetic pathway of vitamin K2 from chorismate. CHA, chorismate; ICHA, Isochorismic Acid; SEPHCHC, 2-Succinyl-5-enolpyruvyl-6-hydroxy-3-cyclohexene-1-carboxylate; SHCHC, 2-Succinyl-6-hydroxy-2,4-cyclohexadiene-1-carboxylate; OSB, 2-Oxosuccinylbenzoate; OSB-CoA, 2-oxosuccinylbenzoyl-coenzyme A; DHNA-CoA, 1,4-dihydroxy-2-naphthoyl-coenzyme A; DHNA, 1,4-dihydroxy-2-naphthoate; DMK, demethylmenaquinone; VK2, vitamin K2. *menF*, isochorismate synthase; *menD*, 2-succinyl-5-enolpyruvyl-6-hydroxy-3-cyclohexene-1-carboxylate synthase; *menH*, 2-succinyl-6-hydroxy-2,4-cyclohexadiene-1-carboxylate synthase; *menC*, O-succinylbenzoate synthase; *menE*, O-succinylbenzoate-CoA ligase; *menB*, 1,4-dihydroxy-2-naphthoyl-CoA synthase; *menI*, 1,4-dihydroxy-2-naphthoyl-CoA thioesterase; *menA*, 1,4-dihydroxy-2-naphthoate prenyltransferase; *ubiE*, ubiquinone/menaquinone methyltransferase. (B) Correlation analysis between the expression levels of intestinal enzymes and the abundance of *C. somerae*.


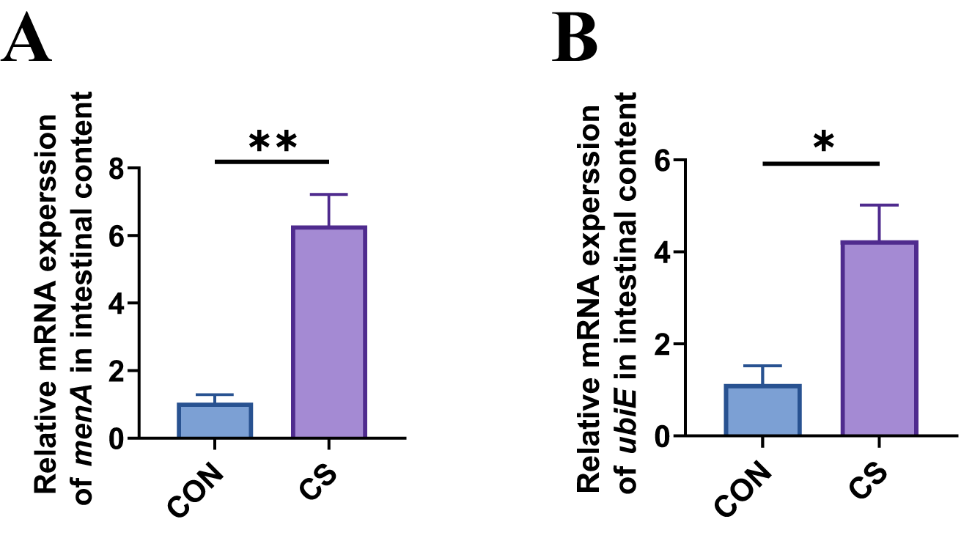


Figure S4. Relative mRNA expression of genes related to VK2 biosynthesis. (A-B) mRNA expression of *menA* (A) and *ubiE* (B) in intestinal content (n=3). * *P*<0.05, ** *P*<0.01. *P*-value was calculated by Student's t-test. *menA*, 1,4-dihydroxy-2-naphthoate prenyltransferase; *ubiE*, ubiquinone/menaquinone methyltransferase.
