# Supplementary figures and images for: Vitamin K2 emerges as the key mediator: Cetobacterium somerae ZNN-1 increases muscle protein deposition and improves liver health in Nile tilapia (Oreochromis niloticus)
Source: J Anim Sci Biotechnol. 2026 Apr 11;17:64. doi: 10.1186/s40104-026-01379-x (PMC13069694; doi:10.1186/s40104-026-01379-x)

**Full uncropped Gels and Blots images**


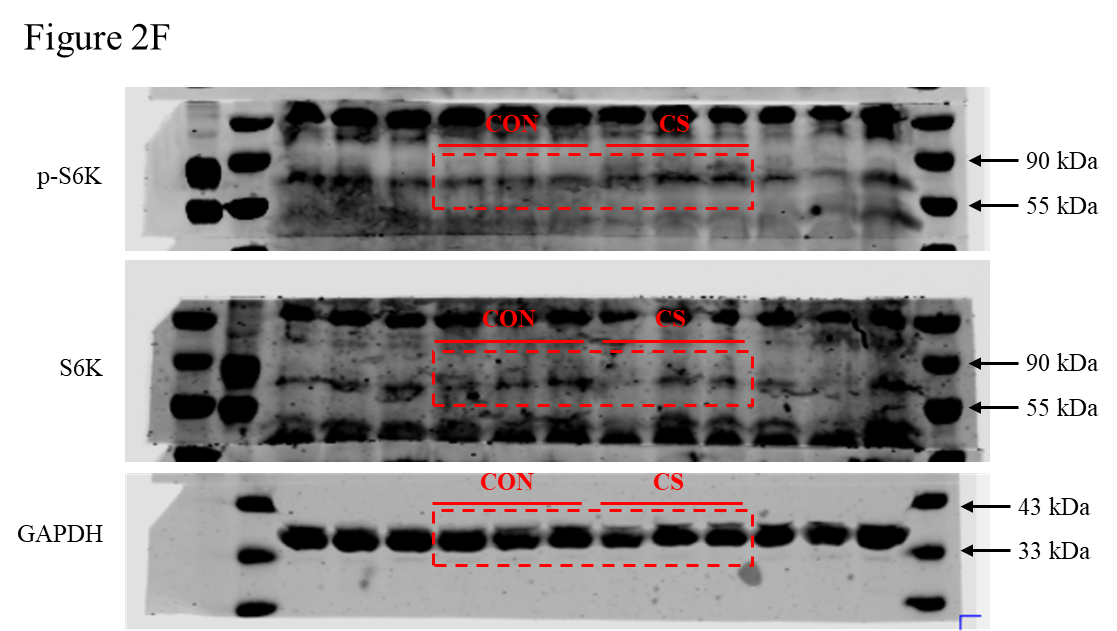


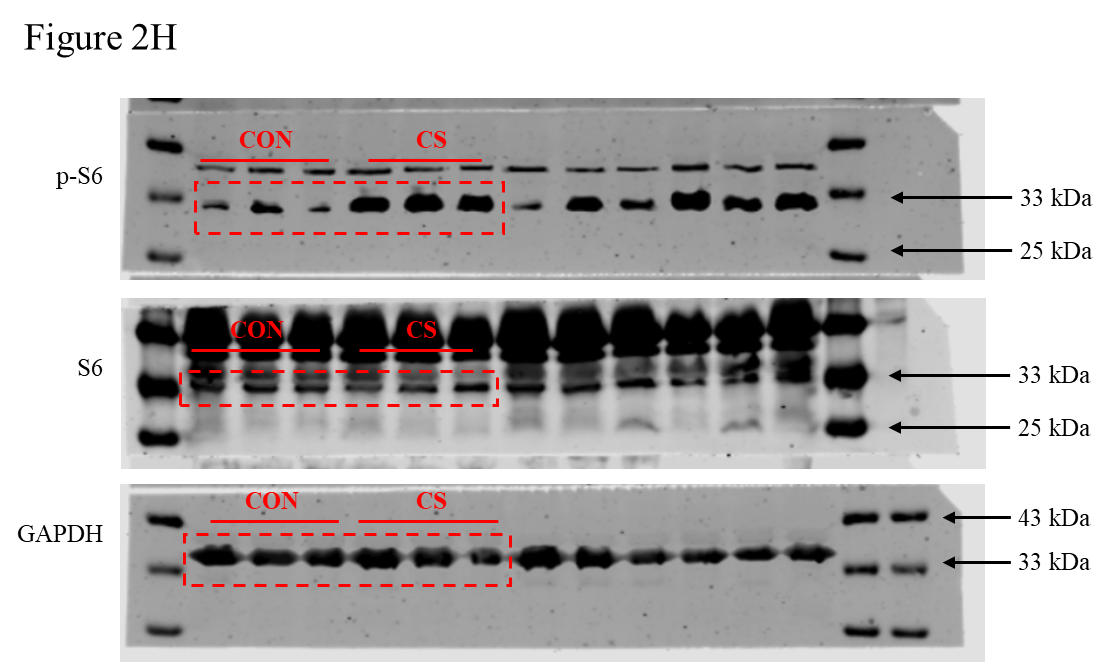


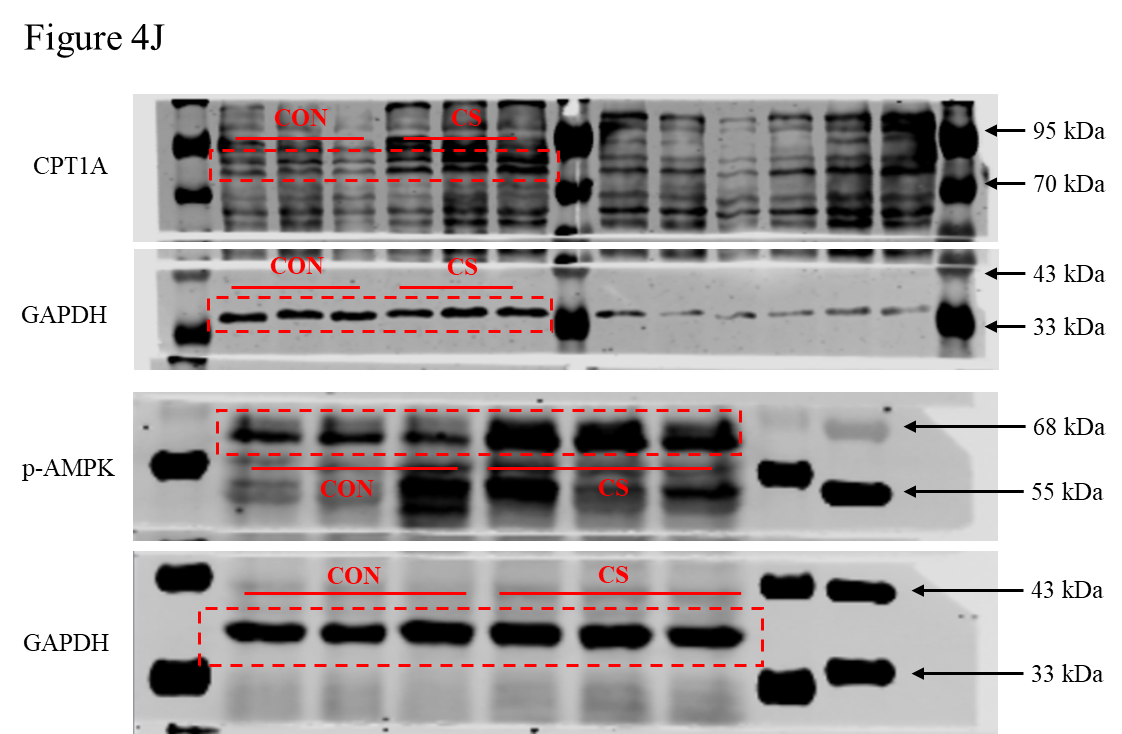


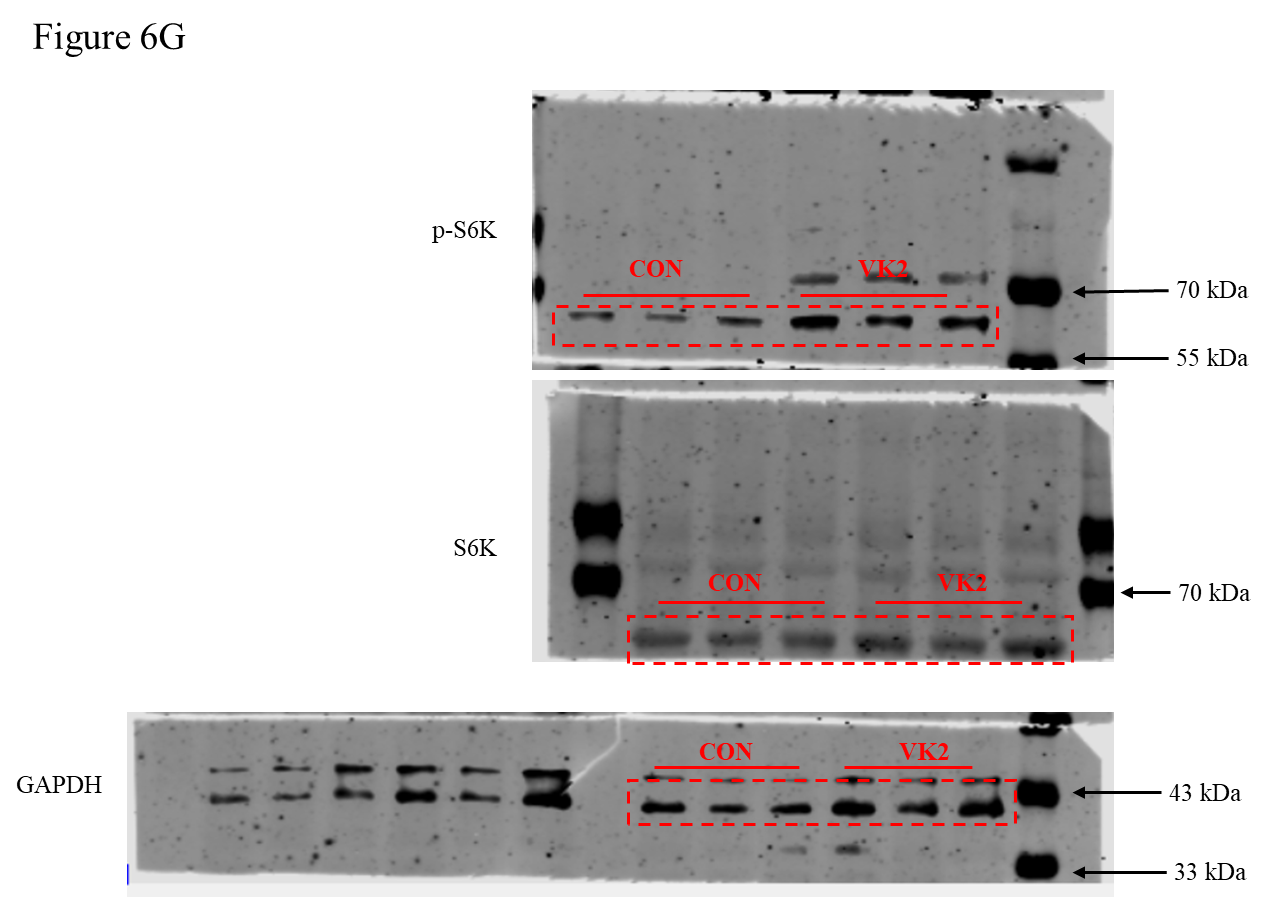


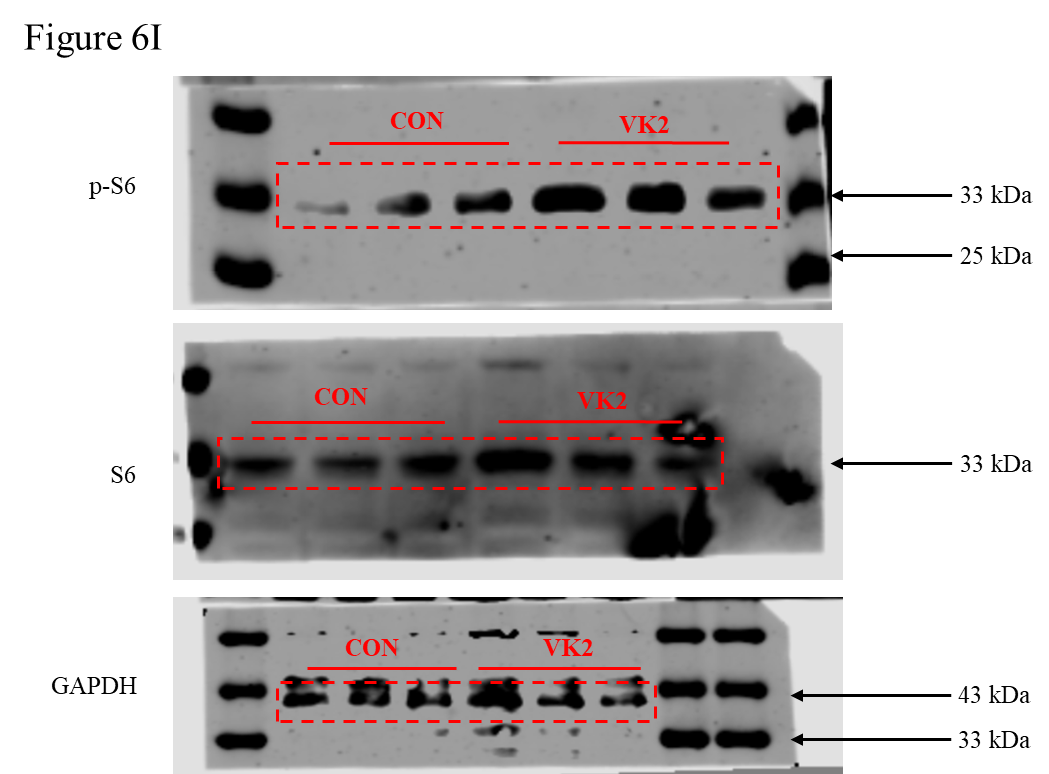


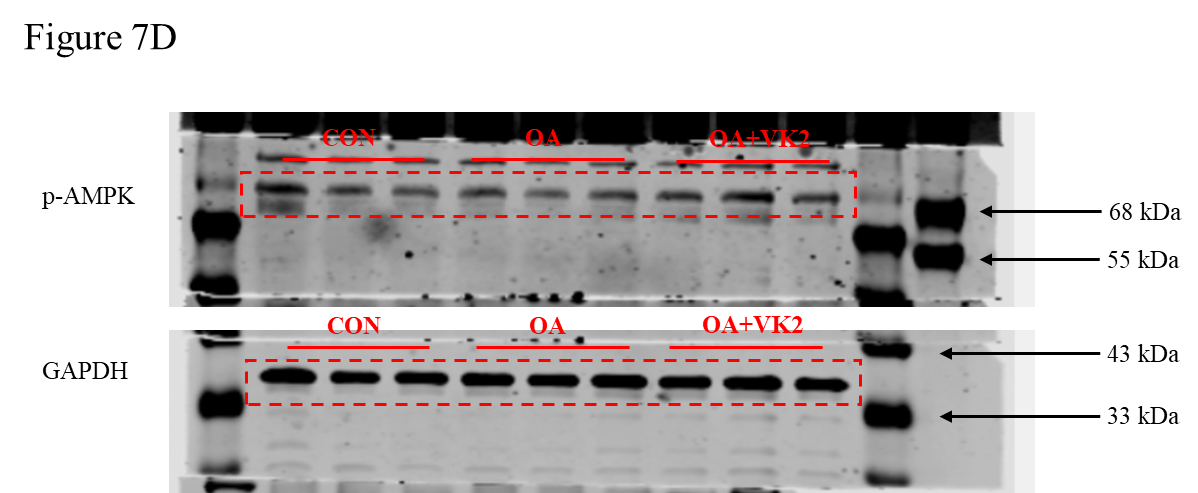

Supplement: Supplementary file 2 — Additional file 2. Full uncropped Gels and Blots images. [file 40104_2026_1379_MOESM2_ESM.docx]
